# Identification of LuxR Family Regulators That Integrate Into Quorum Sensing Circuit in *Vibrio parahaemolyticus*

**DOI:** 10.3389/fmicb.2021.691842

**Published:** 2021-06-29

**Authors:** Xiaojun Zhong, Ranran Lu, Fuwen Liu, Jinjie Ye, Junyang Zhao, Fei Wang, Menghua Yang

**Affiliations:** Key Laboratory of Applied Technology on Green-Eco-Healthy Animal Husbandry of Zhejiang Province, Zhejiang Provincial Engineering Laboratory for Animal Health Inspection and Internet Technology, College of Animal Science and Technology, College of Veterinary Medicine, Zhejiang A & F University, Hangzhou, China

**Keywords:** *Vibrio parahaemolyticus*, quorum sensing, LuxR family regulator, biofilm formation, exopolysaccharide synthesis

## Abstract

*Vibrio parahaemolyticus* is one of the most important food-borne pathogens that cause economic and public health problems worldwide. Quorum sensing (QS) is a way for the cell-cell communication between bacteria that controls a wide spectrum of processes and phenotypic behaviors. In this study, we performed a systematic research of LuxR family regulators in *V. parahaemolyticus* and found that they influence the bacterial growth and biofilm formation. We then established a QS reporter plasmid based on bioluminescence *lux*CDABE operon of *Vibrio harveyi* and demonstrated that several LuxR family regulators integrated into QS circuit in *V. parahaemolyticus*. Thereinto, a novel LuxR family regulator, named RobA, was identified as a global regulator by RNA-sequencing analyses, which affected the transcription of 515 genes in *V. parahaemolyticus*. Subsequent studies confirmed that RobA regulated the expression of the exopolysaccharides (EPS) synthesis cluster and thus controlled the biofilm formation. In addition, bioluminescence reporter assays showed that RobA plays a key role in the QS circuit by regulating the expression of *opaR*, *aphA*, *cpsQ-mfpABC*, *cpsS*, and *scrO*. We further demonstrated that the regulation of RobA to EPS and MfpABC depended on OpaR and CpsQ, which combined the QS signal with bis-(3′-5′)-cyclic dimeric GMP to construct a complex regulatory network of biofilm formation. Our data provided new insights into the bacterial QS mechanisms and biofilm formation in *V. parahaemolyticus*.

## Introduction

*Vibrio parahaemolyticus* is a common marine food-borne pathogen distributed worldwide, which has been recognized as the leading cause of acute gastroenteritis in humans with diarrhea, nausea, vomiting, abdominal cramps, and low-grade fever (Baker-Austin et al., 2018; Ndraha et al., 2020). Although most infections were associated with the consumption of raw or undercooked seafood, septicemia was also reported when open wounds were exposed to this pathogen (Baker-Austin et al., 2018). In recent years, with the global warming and rising seawater temperature, *V. parahaemolyticus* and its epidemic are spreading rapidly around the world (Baker-Austin et al., 2018; Froelich and Daines, 2020).

*Vibrio parahaemolyticus* can adapt to a wide variety of aquatic and marine habitats, which largely depends on quorum sensing (QS) to synchronize bacterial behaviors at the community level, such as stress resistance, motility, and biofilm formation (Yildiz and Visick, 2009; Khan et al., 2020). QS is a form of intercellular communication that enable bacterial populations to solve problems that single bacterial cells cannot, and the extracellular signaling molecules called autoinducers are used as chemical languages by the bacteria to mediate QS (Yildiz and Visick, 2009; Prescott and Decho, 2020). There are three types of autoinducer produced by *V. parahaemolyticus*, including harveyi autoinducer 1, autoinducer 2, and cholerae autoinducer 1 (Zhang et al., 2019). At high cell density, these autoinducers concentrations are high and can be detected by membrane-anchored receptor proteins, which further activates the master QS regulator OpaR to regulates target genes expression (Zhang et al., 2012). At low cell density, OpaR is repressed by another master QS regulator AphA (Sun et al., 2012; Lu et al., 2018). Therefore, OpaR and AphA make a QS circuit to operate QS signal transduction in *V. parahaemolyticus*, which results in the regulation of a wide spectrum of processes and phenotypic behaviors to respond to environmental changes.

Previous studies have reported that many cytoplasmic transcription factors, especially LuxR family proteins, were involved in QS signaling in bacteria (Chen and Xie, 2011). For example, SdiA of *Salmonella typhimurium*, QscR of *Pseudomonas aeruginosa*, and VjbR of *Brucella melitensis* (Ahmer et al., 1998; Chugani et al., 2001; Delrue et al., 2005). These LuxR family proteins contribute to bacterial adaptation to the external environment via binding to autoinducers. At high cell density, autoinducers concentration reaches a threshold, and the LuxR family protein binds to the QS signal and forms an active complex, which further regulates the transcription of target genes by binding to a specific promoter sequence called Lux-box (Chen and Xie, 2011). These LuxR family regulators belong to the homologs of *Vibrio fischeri* LuxR, which are activated when bound to autoinducers. Therefore, LuxR family regulators contribute to bacterial survival, propagation, as well as biofilm formation, and pathogenicity. Understanding the function and mechanism of LuxR family regulators in *V. parahaemolyticus* will promote the development of new treatment strategies for the bacterial induced infections. However, LuxR family regulators remain to be explored and their function is largely unknown in *V. parahaemolyticus*.

Quorum sensing is a complex physiological process and the mechanism is still unclear in *V. parahaemolyticus*. To better understanding the QS mechanisms, we carried out a systematic research of LuxR family regulators in *V. parahaemolyticus*. Through our investigations, we have identified a novel global regulator designated RobA, which regulated the expression of exopolysaccharides (EPS) and MfpABC via QS circuit and constructed a complex regulatory network of biofilm formation. These findings will contribute to a better understanding of the QS mechanisms and biofilm formation in *V. parahaemolyticus*.

## Materials and Methods

### Bacterial Strains and Plasmids

The bacterial strains and plasmids used in this study are summarized in Supplementary Table 1 in the Supplemental Material. *V. parahaemolyticus* strain HZ was used as the wild-type (WT) strain, which was a clinical isolate from the Zhejiang Provincial Center for Disease Control and Prevention (Zhejiang, China) (Yu et al., 2015). *V. parahaemolyticus* was grown at 37°C in Luria-Bertani (LB) broth with 3% NaCl (MLB) containing appropriate antibiotics. *Escherichia coli* strains DH5α, BTH101, and CC118λpir were grown in LB broth with 1% NaCl at 37°C. When required, the culture medium was supplemented with 50 μg/mL streptomycin, 5 μg/mL chloromycetin, 50 μg/mL kanamycin, or 10% sucrose.

### Recombinant DNA Techniques

In-frame deletion mutants of the target gene were constructed using suicide vector pDS132 as described previously (Yu et al., 2015; Wu C. Q. et al., 2019). Briefly, the regions flanking the target genes were cloned into pDS132 vector using specific primers with restriction enzyme sites (Supplementary Table 2). The recombinant plasmid containing a *sacB* counter selectable marker was introduced into *V. parahaemolyticus* or *E. coli* by conjugation, which exchanged genetic fragments twice with the genomes of *V. parahaemolyticus* by intermolecular recombination. Putative deletion mutants were selected using PCR and verified by sequencing.

The pBBR-*lux* reporter plasmid was constructed to check the QS status in *V. parahaemolyticus*. The open reading frame (ORF) of *lux*CDABE operon from the genome of *Vibrio harveyi* was amplified, as well as the putative promoter sequences. These two sequences were ligated by overlap extension PCR, and the fusion fragment was digested with respective endonucleases to build the bioluminescence plasmid pBBR-*lux*. After transformation into *E. coli* DH5α for propagation, the recombinant plasmid was introduced into *V. parahaemolyticus* by conjugation.

### Growth Kinetics Assay

The WT and *luxR* mutant strains grown in the logarithmic phase were diluted to an optical density at 600 nm (OD_600_) of 0.01 in MLB, which was then incubated at 37°C under shaking and static conditions, respectively. The value of OD_600_ was determined at 1-h intervals for 15 h. Each growth curve was derived from at least three independent experiments.

### Biofilm Quantification

Strains in the logarithmic phase were diluted 100 times with MLB, and 2 mL of cultures were added to 3-replicate 5-mL borosilicate culture tubes and incubated statically at 37°C for 24 or 48 h. After incubation, the free-floating bacteria and liquid medium were discarded. The tubes were washed with phosphate-buffered saline (PBS) carefully and stained with 0.04% crystal violet for 20 min. Subsequently, the tubes were washed with PBS, and air-dried for 1 h. The crystal violet was extracted by 2 mL 30% dimethyl sulfoxide (DMSO), and the biofilms were quantified by measuring OD_595_ using the microplate reader (BioTek Instruments, Inc.).

### Bioluminescence Reporter Assay

To identify the bacterial QS status, the pBBR-*lux* reporter plasmid was introduced into *V. parahaemolyticus* by conjugation. Then, the overnight *V. parahaemolyticus* cultures were subcultured with shaking at a dilution of 1:100 in MLB. The value of luminescence and OD_600_ were measured using a Bio-Tek Synergy HT spectrophotometer at 2-h intervals for 15 h. Luminescence expression was reported as light units / OD_600_.

To identify the regulation of RobA to the target genes, the pBBR-*lux* plasmid was rebuilt by replacing the promoter region. The promoter-proximal region of each target gene was predicted by the BProm program (SoftBerry) and amplified using specific primers (Supplementary Table 2). These promoter sequences were ligated with *lux*CDABE operon, respectively, and the recombinant plasmid was introduced into WT and mutant strains by conjugation. The value of luminescence and OD_600_ were measured 12-h post-incubation. Luminescence expression was calculated as above. Each sample procedure was repeated at least three times.

### Bacterial Two-Hybrid System

To analyze the dimerization of RobA protein, we performed the bacterial two-hybrid system to measure the β-galactosidase as described previously (Xue et al., 2016). Briefly, the coding region of RobA was cloned into the pUT-18C and pKT25 vector, respectively, and then both the recombinant plasmids were introduced into *E. coli* BTH101. After incubated statically in LB medium containing 0.5 mM isopropyl-β-D-1-thiogalactopyranoside at 30°C for 8 h, the cultures were assayed for β-galactosidase activity. The BTH101 strain containing the empty pUT-18C or pKT25 vector was used as the negative control.

### RNA Isolation and qRT-PCR Analysis

The strains in the logarithmic phase were washed three times with PBS, and total RNA was purified using TRIzol reagent (Takara) according to the manufacturer’s instructions. Subsequently, the DNase digestion and RNA reverse transcription were performed using the PrimeScript RT reagent Kit with gDNA Eraser (Takara). The quantitative reverse transcription-PCR (qRT-PCR) was performed to validate the transcript concentrations of the selected genes using the Mx3000P PCR detection system (Agilent) and ChamQ Universal SYBR qPCR master mix (Vazyme). All primers specific for tested genes are listed in Supplementary Table 2. The housekeeping gene 16s rRNA was used as an internal control in all reactions (Zhang et al., 2020), and the relative fold change was calculated using the 2^–ΔΔ*CT*^ method. Each sample procedure was repeated three times.

### Transcriptomic Analysis

RNA was extracted from the Δ*robA* and WT strains and further sent to Novogene (Tianjin, China) to generate the transcriptome library, which was sequenced using the Illumina HiSeq 2000 platform as described previously (Zhong et al., 2018, 2019). The transcriptome reads against the reference sequence of strain HZ were mapped by the TopHat2 software, while the differentially expressed genes in the transcriptomic data were identified by the Cuffdiff program. To control the false discovery rate in the transcriptome data, comparisons with estimated fold changes of ≥2 and *q* values of <0.05 were declared significant.

### Statistical Analysis

The data were analyzed using an unpaired two-tailed Student’s *t* test with the GraphPad software package. For all tests, a *P* value < 0.05 was considered statistically significant.

## Results

### LuxR Family Regulators Affect the Growth and Biofilm Formation of *V. parahaemolyticus*

**FIGURE 1:**
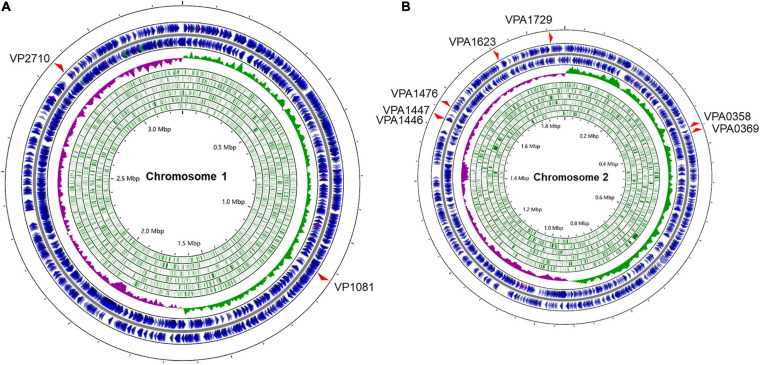
Distribution of LuxR family regulators in the genomes of *V. parahaemolyticus*. The circular diagram depicts the location of 9 LuxR family regulators screened from the P2TF database on the *V. parahaemolyticus* chromosome 1 **(A)** and 2 **(B)**. The outermost blue circles represent the coding sequence (CDS) on the positive or negative chains. The innermost green circles represent the open reading frame (ORF). The middle circle represents the GC skew value and the specific algorithm is (G−C)/(G+C) (positive GC skew, green; negative GC skew, purple). Maps were established using the software CGView Server^*BETA*^ (http://cgview.ca/).

**FIGURE 2:**
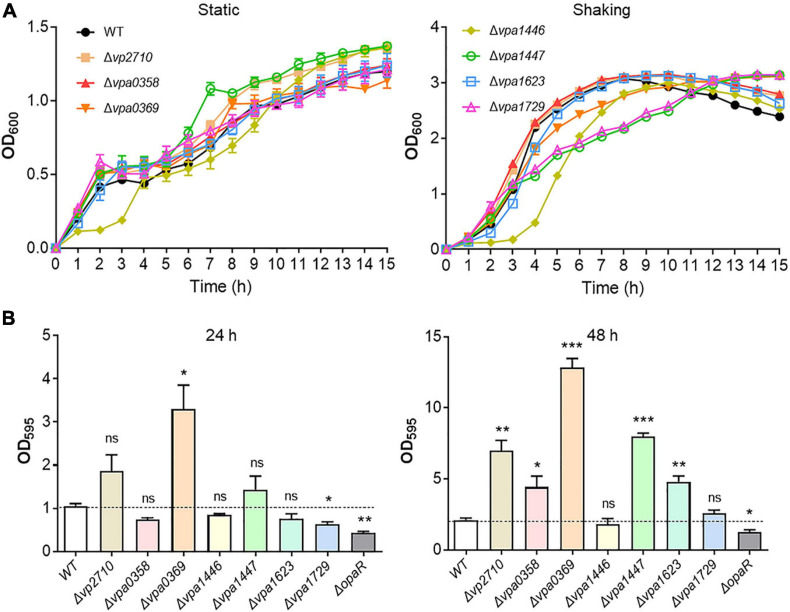
Growth curves and biofilm formation of the WT and *luxR* genes mutant strains. **(A)** The bacterial growth curves of the WT, Δ*vp2710*, Δ*vpa0358*, Δ*vpa0369*, Δ*vpa1446*, Δ*vpa1447*, Δ*vpa1623*, and Δ*vpa1729* strains under the static or shaking condition. Bacteria cell density was measured spectrometrically at OD_600_. **(B)** Biofilm formation of the WT, Δ*vp2710*, Δ*vpa0358*, Δ*vpa0369*, Δ*vpa1446*, Δ*vpa1447*, Δ*vpa1623*, Δ*vpa1729*, and Δ*opaR* strains was measured by crystal violet staining and quantified by measuring OD_595_ at 24 h or 48 h. All data were presented as the means ± SD from three independent experiments. The unpaired two-tailed Student’s *t* test was used for statistical analysis (ns, *P* > 0.05; *, *P* < 0.05; **, *P* < 0.01; ***, *P* < 0.001).

The complete collection of LuxR family regulators were sourced from P2TF database^1^, which provides detailed annotation of each transcription factor including classification, sequence features, as well as functional domains. LuxR family regulators were characterized by their conserved domain architectures GerE (PF00196) or HTH_LUXR (SM00421) in P2TF database, and nine *luxR* genes were detected in the genomes of *V. parahaemolyticus* (Figure 1). We managed to construct the deletion mutant of these *luxR* genes except *vp1081* and *vpa1476*, and then measured growth curves of all these mutants by detecting OD_600_ under static and shaking conditions. Although the growth curves displayed no significant difference among these strains under the static condition, three stains of Δ*vpa1446*, Δ*vpa1447*, and Δ*vpa1729* grew slower than WT strain under the shaking condition (Figure 2A).

Biofilm is a mode of lifestyle chosen by the communities of microorganisms, which contributes to bacterial environmental survival and transmission (Yildiz and Visick, 2009). *V. parahaemolyticus* has the high capacity to mediate biofilm formation during QS signal transduction (Yildiz and Visick, 2009; Papenfort and Bassler, 2016). To evaluate this potential function of these LuxR family regulators, the WT, Δ*opaR*, and *luxR* mutant strains were cultivated 24 or 48 h separately and assayed with the crystal violet stain. We found that Δ*vp2710*, Δ*vpa0358*, Δ*vpa0369*, Δ*vpa1447*, and Δ*vpa1623* exhibited a significant biofilm increase at 48 h compared to the WT strain (Figure 2B). Thereinto, the biofilm formation of Δ*vpa0369* was most obvious, which even produced a more robust biofilm than that of WT strain at 24 h. Meanwhile, the biofilm formation of Δ*opaR* strain was weaker than that of WT strain, which was consistent with the previous report and verified the validity of our results. These results suggested that several LuxR family regulators can synchronize bacterial growth and biofilm formation in *V. parahaemolyticus*.

### LuxR Family Regulators Mediate Bacterial QS in *V. parahaemolyticus*

**FIGURE 3:**
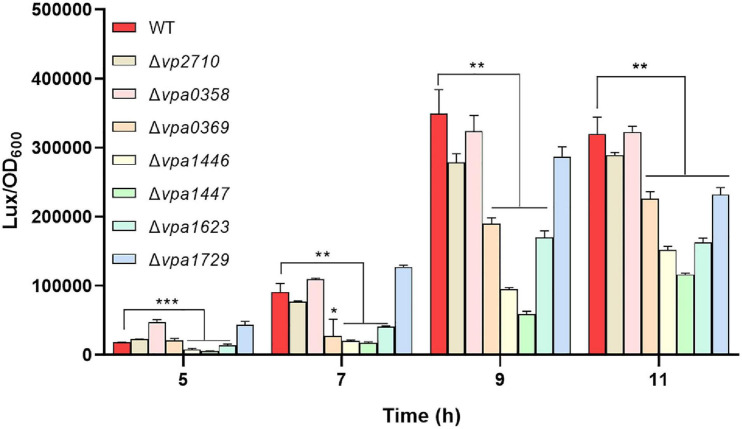
The QS status of *V. parahaemolyticus* was checked by the reporter plasmid pBBR-*lux*. The *luxR* mutants and WT strains were transformed with the pBBR-*lux* reporter, respectively, and the luminescence and OD_600_ were measured at 5, 7, 9, 11 h post-incubation. Luminescence expression was reported as light units / OD_600_. All data were shown as means ± SD from three replicates. The unpaired two-tailed Student’s *t* test was used for statistical analysis (*, *P* < 0.05; **, *P* < 0.01; ***, *P* < 0.001).

To find out the roles that these LuxR family proteins play in regulating QS activity of *V. parahaemolyticus*, we designed a reporter plasmid pBBR-*lux* based on bioluminescence *lux*CDABE operon of *V. harveyi* to detected the QS status in *V. parahaemolyticus* (Supplementary Figure 1A). To determine whether the pBBR-*lux* reporter can be applied to check QS in *V. parahaemolyticus*, the deletion mutant of the master QS regulator OpaR was transformed with the pBBR-*lux* reporter. We then found that the luminescence in Δ*opaR* was significantly weak than that in WT strain after 3 h (Supplementary Figure 1B), which indicated that the luminescence produced from the pBBR-lux reporter was controlled by QS in *V. parahaemolyticus* and verified the effectiveness of the QS reporter. Therefore, the seven *luxR* mutants were transformed with the pBBR-*lux* reporter, respectively, and the luminescence was measured. We observed that the luminescence significantly decreased in Δ*vpa0369*, Δ*vpa1446*, Δ*vpa1447*, Δ*vpa1623*, and Δ*vpa1729* compared with that in WT strain, which suggested that these LuxR family regulators mediate the QS of *V. parahaemolyticus* (Figure 3).

### Transcriptomic Analysis Identifies RobA as a Global Regulator

**FIGURE 4:**
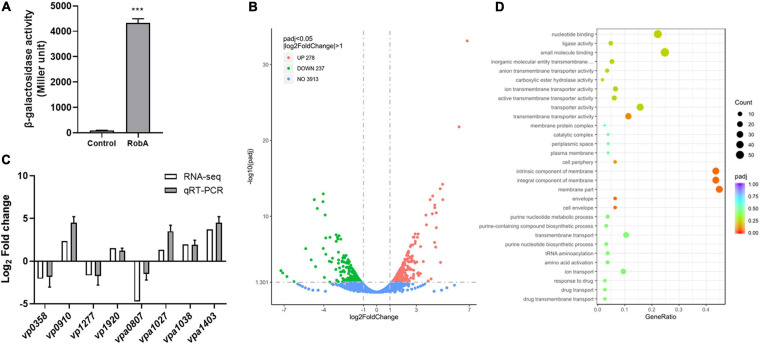
RNA-Seq analysis of RobA-regulated genes. **(A)** RobA dimerization assay in the bacterial two-hybrid system. Full-length RobA was cloned into the pUT-18C and pKT25 vector, respectively, and the empty pUT-18C or pKT25 vector was used as the control. Both the plasmids were introduced into *E. coli* BTH101 to measure β-galactosidase activity, which was reported as Miller Units. The unpaired two-tailed Student’s *t* test was used for statistical analysis (***, *P* < 0.001). **(B)** Volcano plot showing gene expression in Δ*robA* versus WT strains determined based on RNA-Seq analysis. The *x*-axis represents the log_2_ fold change value, while the *y* axis displays the –log_10_(*P*) value. Each dot represented a specific gene. **(C)** Validation of gene regulation by qRT-PCR. Eight genes were randomly selected and amplified by qRT-PCR to validate the expression level change observed by RNA-Seq analysis. All data were shown as means ± SD from three replicates. **(D)** GO analysis of the transcriptomic data. The *x*-axis represents the ratio of the number of differentially expressed genes and the number of all the unigenes in the GO terms. The *y* axis displays the top 30 enriched GO terms.

Bioinformatics analysis indicated that none of the nine LuxR family regulators has the obvious autoinducer binding domain (Supplementary Table 3). Thereinto, VP2710 (ScrP), VPA0358 (ScrO), VPA1446 (CpsQ), and VPA1447 (CpsS) have already been reported in *V. parahaemolyticus* (Güvener and McCarter, 2003; Kimbrough et al., 2020), while VPA0369 remains uncharacterized and would probably be a novel QS regulator. Here we designated VPA0369 as RobA (regulator of biofilm, protein A), and applied the bacterial two-hybrid system to determine whether RobA has the ability of homodimerization. As shown in Figure 4A, RobA can form a homodimer, which suggested that RobA probably behaves as a typical transcriptional regulator. To find out the target genes regulated by RobA, we then performed RNA-Seq and identified that 515 genes were significantly differentially expressed in the Δ*robA* strain compared to that in the WT strain (Figure 4B and Supplementary Table 4).

To verify the reliability of transcriptomes, we randomly selected eight genes of the transcriptomic data to perform qRT-PCR assays. As shown in Figure 4C, the relative expression levels of five upregulated genes and three downregulated genes identified by RNA-Seq were consistent with the qRT-PCR results, which confirmed the changes in RNA transcription levels. Further Gene Ontology (GO) analysis showed that the differentially abundant genes of the transcriptomic data enrich in the categories of biological process, biological process, and molecular function (Supplementary Figure 2). Thereinto, the following terms including the nucleotide-binding and small molecule binding that belong to molecular function categories were significantly enriched (Figure 4D). These results suggested that RobA works as a global regulator and plays important roles in various physiological processes of *V. parahaemolyticus*.

### RobA Regulates EPS Biosynthesis in *V. parahaemolyticus*

**FIGURE 5:**
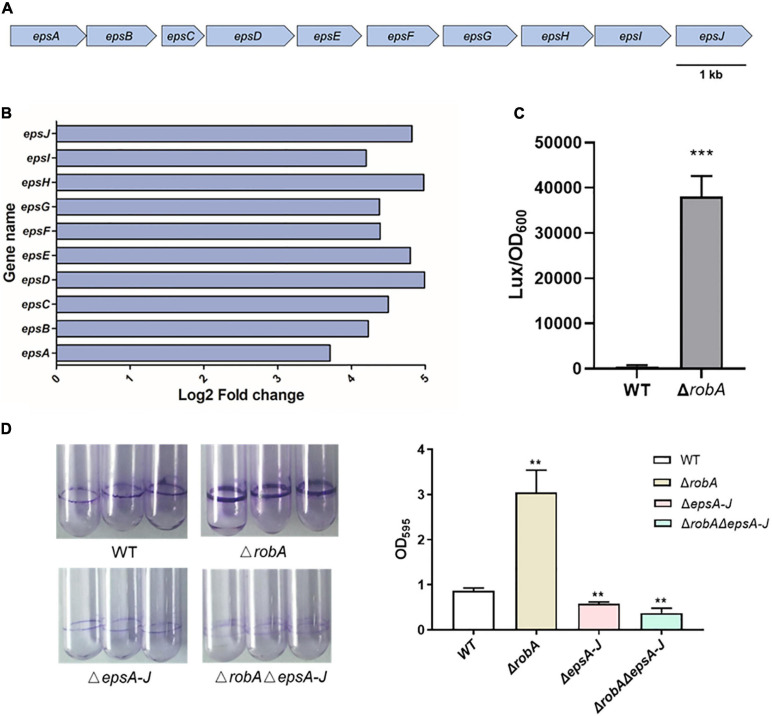
RobA controls EPS biosynthesis in *V. parahaemolyticus*. **(A)** Schematic diagram showing the genetic organization of the EPS synthesis cluster in *V. parahaemolyticus*. The arrows indicate the direction of transcription. **(B)** RNA-Seq analysis showed that *robA* deletion significantly upregulated the EPS biosynthesis clusters. The *x*-axis represents the log_2_ fold change. **(C)** Expression levels of EPS were assessed by measuring luminescence in P*_epsA_*-*lux* transcriptional fusion strains. **(D)** Biofilm formation was measured by crystal violet staining and quantified by measuring OD_595_ at 24 h. All data were presented as means ± SD deviation of triplicate samples from three independent experiments. The unpaired two-tailed Student’s *t* test was used for statistical analysis (**, *P* < 0.01; ***, *P* < 0.001).

The EPS has been proven to be a key chemical component in the biofilm formation of *V. parahaemolyticus*, which forms an intercellular matrix and protects encased bacterial cells (Yildiz and Visick, 2009; Li et al., 2020). The EPS synthesis cluster mainly includes *epsA* to *epsJ* (Chen et al., 2010; Li et al., 2020), and the RNA-Seq analysis showed that the expression level of *epsA-J* was significantly increased in the Δ*robA* strain (Figures 5A,B), suggesting that RobA regulated biofilm formation by controlling EPS biosynthesis in *V. parahaemolyticus*. Subsequently, the promoter region of *epsA-J* was ligated with transcriptional *lux* reporter, which was further introduced into Δ*robA* and WT strain. As shown in Figure 5C, the promoter activity of P*_epsA_*-*lux* was increased significantly in Δ*robA* compared to that in the WT strain. There results indicated that RobA regulated the expression of *epsA-J*. To further figure out the relationship among the RobA, EPS, and biofilm formation in *V. parahaemolyticus*, we construed the *epsA-J* deletion mutant on the basis of the WT and Δ*robA* strain. We found that Δ*robA*Δ*epsA-J* exhibited poor biofilm formation, which was significantly different from that of the single deletion mutants and WT strains (Figure 5D). These results demonstrated that RobA controls biofilm formation by regulating EPS biosynthesis in *V. parahaemolyticus*.

### RobA Integrates Into QS Circuit in *V. parahaemolyticus*

**FIGURE 6:**
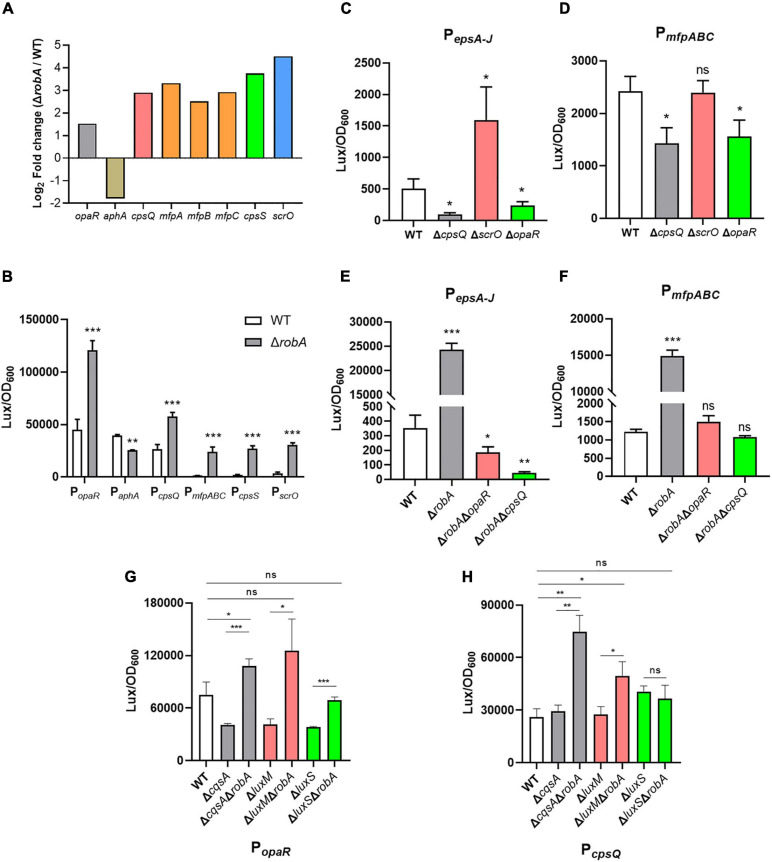
RobA integrates into QS circuit in *V. parahaemolyticus.* **(A)** RNA-Seq analysis showed that *robA* deletion significantly regulated the transcription of *opaR*, *aphA*, *cpsQ*, *mfpABC*, *cpsS*, and *scrO*. The *y* axis represents the log_2_ fold change. **(B–H)** The transcriptional level of *opaR*, *aphA*, *cpsQ*, *mfpABC*, *cpsS*, *scrO*, and *epsA-J*, in *V. parahaemolyticus* were assessed by the bioluminescence reporter ligated with P*_opaR_*, P*_aphA_*, P*_cpsQ_*, P*_mfpABC_*, P*_cpsS_*, P*_*scrO*_*, and P*_epsA–J_*, respectively. Luminescence was measured and reported as light units/OD600. Data are the means ± SD. The unpaired two-tailed Student’s *t* test was used for statistical analysis (ns, *P* > 0.05; *, *P* < 0.05; **, *P* < 0.01; ***, *P* < 0.001).

The QS circuit in *V. parahaemolyticus* was constructed based on the regulatory relationships among OpaR, AphA, and the downstream target genes, which includes the *cpsQ-mfpABC* operon and *cpsS* (Güvener and McCarter, 2003; Sun et al., 2012; Zhang et al., 2012; Zhou et al., 2013). Thereinto, the *mfpABC* encodes the membrane fusion proteins and contributes to biofilm formation in *V. parahaemolyticus*. Here we found that *opaR*, *aphA*, *cpsQ-mfpABC*, and *cpsS*, as well as *scrO*, were all significantly differentially expressed between the Δ*robA* and WT strains (Figure 6A). We then predicted these genes promoter by the BProm program SoftBerry and cloned into the transcriptional *lux* reporter, respectively. The corresponding recombinant plasmids were introduced into Δ*robA* and WT strain to detect the promoter activity. As shown in Figure 6B, when P*_opaR_*, P*_cpsQ_*, P*_mfpABC_*, P*_cpsS_*, and P*_*scrO*_* ligated with the bioluminescence reporter, the promoter activity was increased significantly in Δ*robA* compared to that in the WT strain. Meanwhile, the promoter activity of P*_aphA_*-*lux* was decreased significantly in Δ*robA* compared to that in the WT strain. These results were consistent with the transcriptomic data.

Previous studies have demonstrated that CpsQ, ScrO, and OpaR regulated the EPS or MfpABC expression to contribute to the biofilm formation in *V. parahaemolyticus* (Zhou et al., 2013; Kimbrough et al., 2020). Here we found that both CpsQ and OpaR contributed to the expression of EPS or MfpABC, whereas ScrO acted as a repressor to EPS and not affected the expression of MfpABC (Figures 6C,D). To further investigate the relationship among RobA, OpaR, and CpsQ during the regulation of EPS or MfpABC expression, we created the double genes deletion mutants. We observed that the promoter activity of P*_epsA_*-*lux* and P*_mfpABC_*-*lux* were all decreased significantly in Δ*robA*Δ*opaR* and Δ*robA*Δ*cpsQ* compared to that in the WT and Δ*robA* strains (Figures 6E,F). *V. parahaemolyticus* can produce three types of autoinducer, including harveyi autoinducer 1, autoinducer 2, and cholerae autoinducer 1, which are synthesized by LuxM, LuxS, and CqsA, respectively (Guo et al., 2018; Wu K. et al., 2019). To explore which autoinducer participates in the regulation of RobA to OpaR and CpsQ, we then created a series of genes deletion mutants of the autoinducer synthases. As shown in Figures 6G,H, the promoter activity of P*_opaR_*-*lux* and P*_cpsQ_*-*lux* were all increased significantly in Δ*cqsA*Δ*robA* and Δ*luxM*Δ*robA* compared to that in the WT, Δ*cqsA* and Δ*luxM* strains, respectively. However, the promoter activity was identical in Δ*luxS*Δ*robA* and WT strains (Figures 6G,H), which suggested that autoinducer 2 participates in the regulation of RobA to OpaR and CpsQ. These results indicated that RobA plays a key role in the QS circuit and thus controls the biofilm formation in *V. parahaemolyticus*.

## Discussion

*Vibrio parahaemolyticus* is considered to be an important food-borne pathogen that causes economic and public health problems worldwide (Baker-Austin et al., 2018). QS belongs to the cell-cell communication between bacteria that controls a wide spectrum of processes and phenotypic behaviors in bacteria (Papenfort and Bassler, 2016). Understanding the QS of *V. parahaemolyticus* will help to control and prevent diseases connected to it. In the present study, we performed a systematic research of LuxR family regulators in *V. parahaemolyticus* and demonstrated that they integrated into the bacterial QS circuit, especially the novel global regulator RobA, which plays critical roles in QS signal transduction and biofilm formation.

After firstly characterized by Engebrecht in 1983, LuxR family proteins have been continuously reported due to their important roles in QS signal transduction (Engebrecht et al., 1983; Chen and Xie, 2011). Recently, the LuxR regulator without the cognate autoinducers synthase has been referred to LuxR solo, which is composed of an autoinducer binding domain and a DNA-binding domain (Hudaiberdiev et al., 2015; Subramoni et al., 2015). Therefore, LuxR solo has the ability to bind to QS signal and regulates bacterial adaptation to the external environment. In this study, we have detected nine *luxR* genes in the genomes of *V. parahaemolyticus* using P2TF database, and found that they are functional in many physiological processes. However, further bioinformatics analysis indicated that none of the nine LuxR family regulators has the autoinducer binding domain. Thus, it is possible that the LuxR family regulators have a novel mechanism to sense autoinducers in *V. parahaemolyticus*, or they are in downstream of the regulatory circuit of the bacterial QS. We further found that four of the nine LuxR family regulators have already been characterized, which include VP2710 (ScrP), VPA0358 (ScrO), VPA1446 (CpsQ), and VPA1447 (CpsS). All the four regulators are homologous Scr transcription factors, which can bind bis-(3′-5′)-cyclic dimeric GMP (c-di-GMP) and contributes to biofilm development in *V. parahaemolyticus* (Kimbrough et al., 2020). c-di-GMP is an important second messenger in bacteria, and it could mediate numerous bacterial responses to external conditions, such as EPS production, antimicrobial tolerance, and QS (Valentini and Filloux, 2019; Khan et al., 2020; Petchiappan et al., 2020). With the help of the bioluminescence QS reporter, we observed that CpsQ and CpsS integrate into QS circuit, whereas the ScrP and ScrO are not. This result will help us to better understand their multiple roles in the bacterial physiological processes.

*Vibrio parahaemolyticus* can form biofilms easily on various surfaces, which contributes to the bacterial persistence and becomes a serious problem in food industries (Yildiz and Visick, 2009). Previous studies have identified many key factors that involved in biofilm formation in *Vibrio* species, such as EPS, flagella, QS, c-di-GMP, and the regulators that control their expression (Yildiz and Visick, 2009; Belas, 2014). However, biofilm formation is a complex physiological process and the mechanism is not yet understood, especially the regulatory network during the adaptation to the harsh environments. Our results showed that Δ*robA* has the most obvious phenotypes among these LuxR family regulators mutants, as it forms a robust biofilm. Considering that RobA has never been reported and is a novel LuxR family regulator, we applied RNA-Seq to investigate the molecular mechanisms whereby RobA works in *V. parahaemolyticus*. RNA-Seq analyses combined with bioluminescence reporter assays identified that RobA regulates transcription of the EPS biosynthesis cluster. For numerous *Vibrio* spp., EPS has been shown to function as extracellular matrix components that hold the cells together and aid in adhesion to the surface (Yildiz and Visick, 2009; Li et al., 2020). We further constructed the *epsA-J* deletion mutant and demonstrated that the thick EPS mediated by RobA contributes to the robust biofilm in *V. parahaemolyticus*.

**FIGURE 7:**
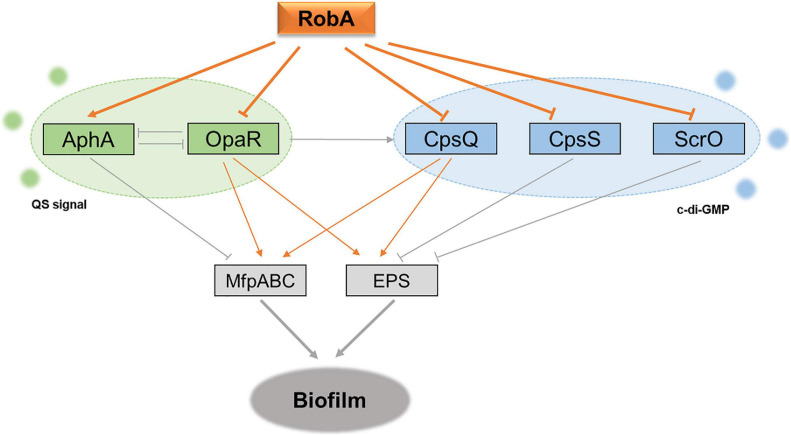
Schematic of RobA in control of QS and biofilm formation in *V. parahaemolyticus.* The gray lines show the regulatory relationships that have been reported in previous studies, whereas the orange represents the relationship identified in this study. AphA and OpaR are the two master QS regulators, and they operate QS signal transduction in *V. parahaemolyticus*. CpsQ, CpsS, and ScrO are the Scr transcription factors, which can bind the second messenger c-di-GMP and further mediate the bacterial EPS production. RobA acts as a global regulator in the regulatory circuit to synchronize the bacterial biofilm formation in *V. parahaemolyticus*, which relies on the repression to the master QS regulator OpaR and Scr transcription factor CpsQ.

OpaR and AphA are the two sole master regulators of QS in *V. parahaemolyticus*, which control hundreds of target genes during QS signal transduction and further form a QS circuit (Sun et al., 2012; Zhang et al., 2012). In the present study, we observed that their transcription levels were repressed and enhanced by RobA, respectively. In addition, the QS reporter plasmid indicated that RobA mediates the QS status of *V. parahaemolyticus*. These results suggested that RobA integrates into the bacterial QS circuit. Further RNA-Seq analyses and bioluminescence reporter assays showed that RobA regulated the transcription of *cpsQ*, *mfpABC*, *cpsS*, and *scrO*. MfpABC is a putative membrane fusion transporter, and CpsQ can activate the expression of EPS and MfpABC (Enos-Berlage et al., 2005; Ferreira et al., 2012; Srivastava and Waters, 2012). The *cpsQ-mfpABC* locus can be transcribed as *cpsQ-mfpABC* and *mfpABC*, and both of them are required for biofilm formation in *V. parahaemolyticus* (Ferreira et al., 2012). Previous studies have shown that OpaR and AphA can enhance and repress the transcription of *mfpABC* and *cpsQ-mfpABC*, respectively (Zhou et al., 2013). CpsS acts as a negative regulator of EPS biosynthesis cluster, and ScrO is the primary regulator of biofilm (Güvener and McCarter, 2003; Kimbrough et al., 2020). However, here we found that ScrO appears to act as a repressor to EPS and biofilm formation in *V. parahaemolyticus* strain HZ, as the deletion mutant of *scrO* has greatly elevated *epsA-J* transcription and biofilm formation, which may attribute to the physiological differences among different *V. parahaemolyticus* isolates. We further demonstrated that the regulation of RobA to EPS and MfpABC depends on OpaR and CpsQ, which suggests OpaR and CpsQ have priority over AphA, CpsS, and ScrO for the biofilm regulation in the RobA network. All the CpsQ, CpsS, and ScrO belong to Scr transcription factors, and their activities are dependent on c-di-GMP (Kimbrough et al., 2020). Therefore, RobA with the target regulators build a new QS circuit that combined the autoinducer with c-di-GMP signals in *V. parahaemolyticus* (Figure 7), which primes the bacteria for environmental adaptability.

In summary, the LuxR family regulators that integrate into the QS circuit have been identified in *V. parahaemolyticus*. Thereinto, a novel regulator RobA was confirmed to play critical roles in QS signal transduction and biofilm formation of *V. parahaemolyticus*, which constructs an adaptable regulatory network for bacteria synchronizes behaviors during the colonization and transmission. However, the underlying mechanisms that the autoinducers or upstream regulators activate RobA remain unknown and require further exploration in our future work.

## Data Availability Statement

The datasets generated for this study can be found in the NCBI PRJNA720252 (http://www.ncbi.nlm.nih.gov/bioproject/720252).

## Author Contributions

XZ, RL, and MY designed the study. RL, FL, JY, JZ, and FW performed the experiments. XZ and MY analyzed the results and wrote the manuscript. All authors contributed to the article and approved the submitted version.

## Conflict of Interest

The authors declare that the research was conducted in the absence of any commercial or financial relationships that could be construed as a potential conflict of interest.

## Footnotes

1 http://www.p2tf.org/
