# Supplementary material for: Identification of LuxR Family Regulators That Integrate Into Quorum Sensing Circuit in Vibrio parahaemolyticus
Source: Front Microbiol. 2021 Jun 29;12:691842. doi: 10.3389/fmicb.2021.691842 (PMC8276238; doi:10.3389/fmicb.2021.691842)
Supplement: Supplementary file 1 [file Data_Sheet_1.pdf]

Supplemental Information for:

Identification of LuxR family regulators that integrate into quorum sensing circuit in *Vibrio parahaemolyticus*

Xiaojun Zhong[#], Ranran Lu[#], Fuwen Liu, Jinjie Ye, Junyang Zhao, Fei Wang, Menghua Yang[*]

College of Animal Science and Technology & College of Veterinary Medicine of Zhejiang A&F University, Key Laboratory of Applied Technology on Green-Eco-Healthy Animal Husbandry of Zhejiang Province, Zhejiang Provincial Engineering Laboratory for Animal Health Inspection & Internet Technology, Hangzhou, China

Supplemental Table S1. Bacterial strains and plasmids used in this study

Strains or plasmids	Description	Source or reference
Bacterial strains		
HZ	*V. parahaemolyticus* clinical isolate, wild type (WT)	(Yu et al., 2015)
Δ*vp2710*	Deletion mutant of *vp2710* with HZ background	This study
Δ*vpa0358* (Δ*scrO*)	Deletion mutant of *vpa0358* with HZ background	This study
Δ*vpa0369* (Δ*robA*)	Deletion mutant of *vpa0369* with HZ background	This study
Δ*vpa1446* (Δ*cpsQ*)	Deletion mutant of *vpa1446* with HZ background	This study
Δ*vpa1447* (Δ*cpsS*)	Deletion mutant of *vpa1447*with HZ background	This study
Δ*vpa1623*	Deletion mutant of *vpa1623* with HZ background	This study
Δ*vpa1729*	Deletion mutant of *vpa1729* with HZ background	This study
Δ*epsA-J*	Deletion mutant of *epsA-J* with HZ background	This study
Δ*robA*Δ*epsA-J*	Deletion mutant of *robA* and *epsA-J* with HZ background	This study
Δ*opaR*	Deletion mutant of *opaR* with HZ background	This study
Δ*robA*Δ*opaR*	Deletion mutant of *robA* and *opaR* with HZ background	This study
Δ*robA*Δ*cpsQ*	Deletion mutant of *robA* and *cpsQ* with HZ background	This study
Δ*cqsA*	Deletion mutant of *cqsA* with HZ background	This study
Δ*cqsA*Δ*robA*	Deletion mutant of *robA* and *cqsA* with HZ background	This study
Δ*luxM*	Deletion mutant of *luxM* with HZ background	This study
Δ*luxM*Δ*robA*	Deletion mutant of *robA* and *luxM* with HZ background	This study
Δ*luxS*	Deletion mutant of *luxS* with HZ background	This study
Δ*luxS*Δ*robA*	Deletion mutant of *robA* and *luxS* with HZ background	This study
E. coli DH5α	Cloning host for maintaining the recombinant plasmids	(Hanahan, 1983)
E. coli CC118λpir	Mobilization of plasmids into *V. parahaemolyticus*	(Yu et al., 2015)
E. coli BTH101	Two-hybrid system detected strain	(Karimova et al., 1998)
Plasmids		

pDS132	Suicide vector for *V. parahaemolyticus* mutagenesis	(Philippe et al., 2004)
pDS132-*vp2710*	Derived from pDS132 used to knock out *vp2710* in HZ	This study
pDS132-*vpa0358*	Derived from pDS132 used to knock out *vpa0358* in HZ	This study
pDS132-*vpa0369*	Derived from pDS132 used to knock out *vpa0369* in HZ	This study
pDS132-*vpa1446*	Derived from pDS132 used to knock out *vpa1446* in HZ	This study
pDS132-*vpa1447*	Derived from pDS132 used to knock out *vpa1447* in HZ	This study
pDS132-*vpa1623*	Derived from pDS132 used to knock out *vpa1623* in HZ	This study
pDS132-*vpa1729*	Derived from pDS132 used to knock out *vpa1729* in HZ	This study
pDS132-*epsA-J*	Derived from pDS132 used to knock out *epsA-J* in HZ	This study
pDS132-*opaR*	Derived from pDS132 used to knock out *opaR* in HZ	This study
pDS132-*cqsA*	Derived from pDS132 used to knock out *cqsA* in HZ	This study
pDS132-*luxM*	Derived from pDS132 used to knock out *luxM* in HZ	This study
pDS132-*luxS*	Derived from pDS132 used to knock out *luxS* in HZ	This study
pBBR-*lux*	The QS reporter containing *lux*CDABE operon and Lux box of *V. harveyi*	This study
P_{epsA}-*lux*	pBBR-*lux* containing the promoter of *epsA-J*	This study
P_{opaR}-*lux*	pBBR-*lux* containing the promoter of *opaR*	This study
P_{aphA}-*lux*	pBBR-*lux* containing the promoter of *aphA*	This study
P_{cpsQ}-*lux*	pBBR-*lux* containing the promoter of *cpsQ*	This study
P_{mfpABC}-*lux*	pBBR-*lux* containing the promoter of *mfpABC*	This study
P_{cpsS}-*lux*	pBBR-*lux* containing the promoter of *cpsS*	This study
P_{scrO}-*lux*	pBBR-*lux* containing the promoter of *scrO* operon	This study

Supplemental Table S2. Primers used in this study

Primers	Primers sequence (5’-3’)
vp2710 deletion	P1: GCGTCTAGACTGATTGTGAGAGTTGGAAG

	P2: CAAGCAATGGCTTGCATACTAATCAGAAATAGCTTTCT
	P3: CAAGCCATTGCTTGGGCCGATCAAAACTTGAT
	P4: GCGGAGCTCTTTTGCTTGTTTCAGCAGCGC
vpa0358 deletion	P1: GCGTCTAGACTAAACATCAATCCTCGTTTT
	P2: ACCAACAACACCATTACGTGGGCAAAACGTAAT
	P3: TGCCCACGTAATGGTGTTGTTGGTTGTGTTGG
	P4: GCGGAGCTCTCGCTTTCCTCATGAAGGAA
vpa0369 deletion	P1: GCGTCTAGACGGTTTACCTTGGCTTGCT
	P2: CAACTCGCAGAACAATTGTTCCTCAACCACCT
	P3: GAGGAACAATTGTTCTGCGAGTTGCGCGTTAA
	P4: GCGTCTAGAACCTCTCCTGCTCATGCA
vpa1446 deletion	P1: GCGTCTAGAGGGATAGCAAGTACGAGTA
	P2: ATGGAACAGTACAGTTGGGCAAAAAGAAACCT
	P3: TTTTTGCCCAACTGTACTGTTCCATAATGTTTCT
	P4: GCGGAGCTCAAACTACTAAAGGAATGCGA
vpa1447 deletion	P1: GCGGAGCTCTTACATGCAGTTATTCCTAGTAC
	P2: ACTACAAGGAATTCAGATCACTCTATAGAGATG
	P3: AGAGTGATCTGAATTCCTTGTAGTTTGATGCT
	P4: GCGGAGCTCGATTGAAGCGGCACGCGCAG
vpa1623 deletion	P1: GCGTCTAGATCGCTTGTTGGATGCGAGGA
	P2: AGTTACAATGGCGGCATTGTGTAGTCGGCCGGG
	P3: CTACACAATGCCGCCATTGTAACTGCGGAGAAT
	P4: GCGGAGCTCCATCGCGTTTCTCTACCTGA
vpa1729 deletion	P1: GCGTCTAGAACAATCTGCGTGTAAGCATGA
	P2: ATTCAAAACAACTACTGCTATTTGGCCCCA
	P3: CAAATAGCAGTAGTTGTTTTGAATGGAGAGCG

	P4: GCGTCTAGATTATTTTGGGATGACTTCACG
epsA-J deletion	P1: CTAGTCTAGAATACCTAAACCTAGAAGGCGCAGACG
	P2: ATCGTTCTAAGTAGGGCGCTTGTCATTAACC
	P3: GCGCCCTACTTAGAACGATGAAATACATCACGATTTGTTC
	P4: GCATGCATGTTTTGGCAATTCGATGGCTTCTTGTTC
opaR deletion	P1: GCGTCTAGATCGCGATAGATTTAGGTTCAC
	P2: TCTAGGTAAGCGCTTTGTTGCTTACGTTTAAGAG
	P3: AGCGCTTACCTAGATATGCTTTGCATCTAC
	P4: GCGGAGCTCAATCGACGAGCGCGGCTTCATCA
luxM deletion	P1: GCTCTAGACCTACAGATCCTAGTATTCTGTATGTTCTT
	P2: CCCAACCTTGTAAAGCGAAACCAAAGACAG
	P3: TTGGTTTCGCTTTACAAGGTTGGGTTCACC
	P4: CGAGCTCGCAAGGTATTTGGCGCTGTAGAATCTCGAT
luxS deletion	P1: GCTCTAGATTCCTACGCGAGCAGCATCTTGCTCACTAT
	P2: TTCACCGTAGACTCAATGCTAAAAGAGCTGCGC
	P3: TTTTAGCATTGAGTCTACGGTGAAGCTATC
	P4: CGAGCTCCAAGCTAGAAGCATGGATTGATAACCCTGA
cqsA deletion	P1: ACATGCATGCGCCGGGAGAGTGGTCACAGTTTACGTTGAC
	P2: CGCCTTAATTTCGATCACGTCTTATCGGCT
	P3: TAAGACGTGATCGAAATTAAGGCGTTCCTCGA
	P4: AACTGCAGGGGCATCCCAAACGTGGGTAAATCGACCAT
pBBR-*lux*	P1: CGCGGATCCGCGCATCGTTAGGAGCTCCTACTTA
	P2: CGCGGATCCATCAAGAGCTTCTCCTTTGAATTTG
P_{epsA}-*lux*	P1: GCGGAGCTCCTTTTCCTCATCCCTGCTTA
	P2: GCGGGATCCGACCTAGTTTCCCTTCTAGCA
P_{opaR}-*lux*	P1: GCGGAGCTCTAGTGTATCACCACGAAGAG

	P2: GCGGGATCCATCCATTTTCCTTGCCATTT
P_{aphA}-*lux*	P1: GCGACTAGTAACCCATAAAATACCAACAAAT
	P2: GCGACTAGTGTCTTCAATCCAAATGGTCA
P_{cpsQ}-*lux*	P1: GCGGAGCTCAGATCACTCTATAGAGATGG
	P2: GCGGGATCCACTTTCATTAACTTAAGATTCTTAATT
P_{mfpABC}-*lux*	P1: GCGGAGCTCAAAATAGGACGCAAGCCACA
	P2: GCGGGATCCTTTTATTCCCTCTGGCTTAT
P_{cpsS}-*lux*	P1: GCGGAGCTCCTGTTCCGGTTCATTAATAAA
	P2: GCGGGATCCCCTGCTCGCATTCCTTTAGT
P_{scrO}-*lux*	P1: GCGGAGCTCAAAACCGTATAGTTTTATGCC
	P2: GCGGGATCCTTAGGAACAGCAAATAATGGG
pUT-18C/pKT25-VPA0369	P1: GCGGGATCCCATGCCTAACCAAAACTTTA
	P2: GCGGGTACCCTATTTTGAGTGCATGATCAG
qPCR-*vp0358*	P1: GCCATCCCTACGGAAGTTATT
	P2: CTACCGCTGTCTATCACGATTC
qPCR-*vp0920*	P1: AAGGCAACTACCGTCAAGAG
	P2: ACCAGCTAACTCACCCAAAG
qPCR-*vp1277*	P1: CGGACGATAGCATCGCAATAA
	P2: CTGTTTACCCAACGCAGTTCTA
qPCR-*vp1920*	P1: AAGGCAACTACCGTCAAGAG
	P2: ACCAGCTAACTCACCCAAAG
qPCR-*vpa0807*	P1: CGTTGCTGACAGGGTGTAATA
	P2: GTCAGAGTCGTCAACCGTAAG
qPCR-*vpa1027*	P1: AGCGACAGCGGAACAATATAA
	P2: CTTCACGGTCCATTGCTGTA
qPCR-*vpa1038*	P1: GGGTGACACTTGGAACGATTA

		P2: TGGAATCATGCTGCCGATATAC
qPCR-*vpa1403*		P1: CAAGTGATGAGCATGCAAAGG
		P2: GCCGTGAGTCCCAGAAATAAc
qPCR-16s rRNA		P1: AAGCGTGGGGAGCAAACAG
		P2: CGAAGGCACCAATCCATCTC

Supplemental Table S3. Protein sequence and domain architecture of the 9 LuxR family regulators

Gene locus	Protein sequence	Domain architecture
VP1081	MIVVNPLRKCKLTGELYRRLPEIESKLSELLQLPEETLVERSLINDKTSVQFVPIECLVHIVRNVGG<br>RASKKSYERLYKILMGRVLKLIPKRATRGDLDSVTNTEVKSQILGQFAELIANDCLEYNDKLDFY<br>EVRFLSAFSTLKTDAIRKVTKGFTKQESMEVEESGGIIKPEVEYAVDGYNPFDVHISSVSDYQIYL<br>DSAIDTLPDLQKRIMQLMKLGMPIDSKDPNTESISATLGKSEKTIRTHRNKAFAALKKKLTGGDL	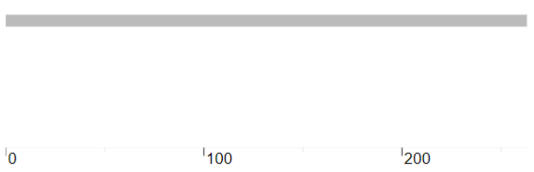
VP2710	MRKSAYARKLFLISMEDDAAQKVASLEKYIDMSIPVISTDALMEAKPEHRNKILLIDFSEHKSLV<br>QSIKNLPLVWKNFETVVFNVPKRLTTDELLAFGQLKGLFYSEDSLEQVGEGLKGIVNGQNWLPR<br>NVTSQLLHYYRNVINTHTAPATVDLTIRELQVLRCLQAGASNSQMAEELFVSEFTIKSHLYQIFK<br>KLSVKNRVQAIAWADQNLMS	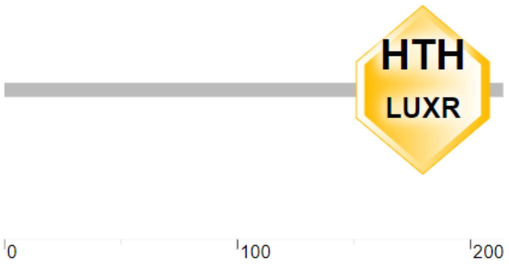

VPA0358	MMNTNTTNNTVLLFTFNNIQGRGLQTAIEQNLEKPVLLTQGADVNLPSTRDEHYVAIVDSSLPEL<br>PEVKQTLSELEHVDATVLVNAEPNLRIESLLTWSNLKGLFYFEDDFDKVMMGLKGILNGENWL<br>SRDILNQLIGHLLSLNNTVGELETKLEMELTRREMQVLSALCQGGSNLDIADSLFVSEHTVKSHL<br>YSIFRKLEVKNRMQAITWAKRNLL	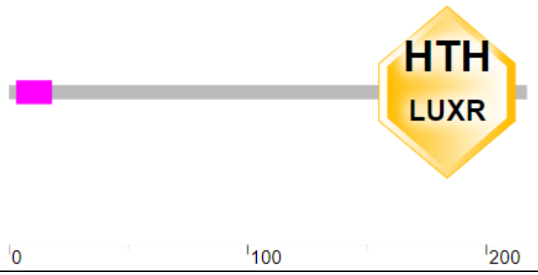
VPA0369<br>(RobA)	MPNQNFNAQLAEAISALNTPNFTPKLMSVIHSIFDFDCAIILGYREGKHPIYLYDSIENERELLFQR<br>YLTNSFQNDPFFQNLNQHKQQGIFTLKDVAKKGIEYQTYRKQFYDQTGWKDELSMLVEIESGR<br>WVILYFGCLREGKRFSAAQINNLRSHFSVLQSLCQQHWKQAEFNLSEPVVRPDAYTGNMKVAIE<br>QALSSFGIESLTRREQEVASLLAQGFDTKEISAHLHLVQGTVKNHRKRIYSQLNVSSLSELFQLFL<br>NHLIMHSK	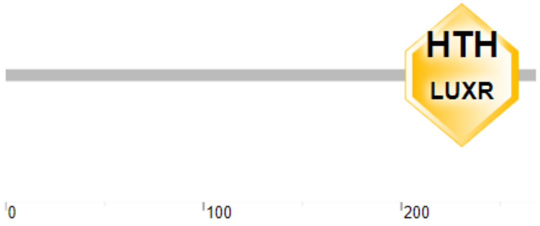
VPA1446	MRNIMEQYTEKPEILMLTQQSLQSENFKEMLSKNTETKITIIDAKNPSYHELIPDRYFLLVDFSVD<br>TPSDTLVYLKDSNKVLGTIMLNLGYDLDTEELASWPHVKGIFGPLDSMEKVCRGLGAIVKGDN<br>WLSRRLLDQLVNYYKGKESNNVSEPAIEVELTRREIQVLKMLKEGGSNMEIADSLFISEHTIKSH<br>LYNIFRKLEVKNRTQATSWAKRNL	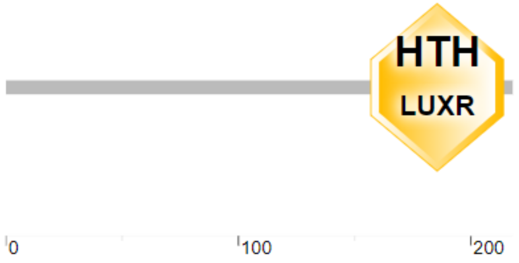

VPA1447	MEHQTTRNVILITEGSLQSSLLKDVLETKLGINVLLITPENLASPFVRNQSISAIVLDYSVITDEVF<br>ARYMEFKTPNLTGTLEILINCDKSISTDELFVWGALAGIFYTSDDIQTLQTGIDKVLQGDMWFSR<br>KFAQQYITHLRRHSRPINKNVPAILTKREQQIITFLSMGASNQQIAEQLFVSENTVKTHLHNIFKKI<br>DVKNRVQALIWAKENISDHSIEMV	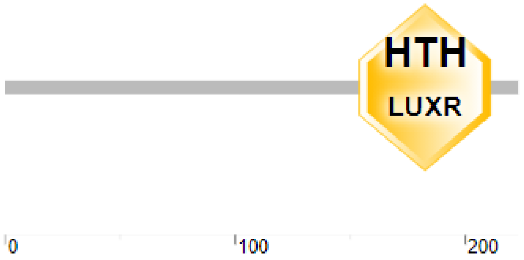
VPA1476	MSNGELPKDADGLQLNFCKTLACDNFGLSDAKRYVLQHANPKRPAMVCRECGAFPPLLNNRD<br>VVNELHRLRHVHSDGLPACRNDACDNFGLSVHTHKHLYHAFGYSGDRQRYRCKACQSTFVDK<br>WSGANKKLQFQENLMGLLFTGYSVREICRKLSINPKTFYDHVDHIASRCRRKLATIDARWVNH<br>ASHYELASSYIALQPHSNNGVYWIVSGEAHSGYILCQHVNYSSDEEPIATLDHNPYDEVSRFVSQ<br>EYTAEASEPPAEPSNHLRERIDQKYQTILARGNVEDPLGNLSVFHYPSKGALVRPPYTSYAHYLH<br>VLDMCCPNKRVSIYMPQDPLLRSAALSVCLSRIQEKNVDLMYVEEDAGWDMTAPFGKVDIAY<br>MSWWRDRWAISSQGESHKGICYLAGDKNEPEKWFNVATTRHVQFYQNRFQLLFESFINEPRRK<br>LRPAGILPLLDIFRAWHNLCYQDKQGLTAAQRLEVTDAPLTIKQLLS	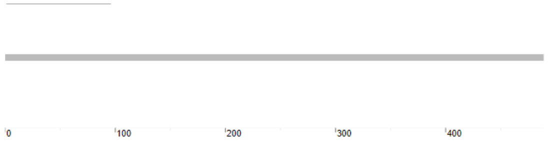
VPA1623	MWIPSKLTRPGRLHNAIVRPRVLDLLQQAPYYKLVLFRSPAGYGKTTMAAQWLSDKPNVGWY<br>SIDDSDNDGFRFVNYLLQALNKATNFSCSNAQKLAEKRQISSLRSLFSEVFAEMADFHQECYVV<br>LDDYHLITNDEIHESMRFFLKHMPDNLTVVVTSRAAPPLGTANLRVRDLMIEIGNEMLAFDTEET<br>TRFFNQRIADGIDEDMANSLRTYVEGWPSAMQLIALQAQHQNRTLAQTVESVSQFNHAHLWDY	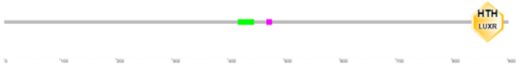

	LVEEVFDLLDHETRHFLMQVSVLDHFNDELVFALTQREDALGLIESLNRYGLFIYPLEGEHNWFR<br>FHNLFGEFLSHERQARIPQQEKDLHRNAAVAWLQQKSPHQAIHHAQKSNDKDLVVEILNEFGW<br>KMFNQGELSTLEHAINKLDAELLFSHPKLTMLRAWLAQSQHRYNQVGQLLEEAEEEHKKRNIE<br>LDIHYQGQANALLAQVAINSNQPEKALELAELALSQLDNTIYRSRIVATSVVGEVNHVLGKLDR<br>ALPMMQQTEKLARQYQVYHQALWAILQQSEILIAQGYVQAAFELQDSGFRLIEDQQLQHVPLH<br>EFLLRIRAQVLWCWNRLDEAEECAYRGLQILENHSPSKHLHSYSMLARIAIGRGELDKAGKFIE<br>HIQHLMKQSTYHVDWTANASLSLILFWQARGNTEAMQEWLNTAVRPESACNHFLQLQWRNIV<br>RAHINLGQYEEARQALNFLQSEARRTNLITDTNRNLVVEAVLAARQKDEEQAKALLKEALVMT<br>NQTGMVGNFLIDGATIGGLLEKLSLRHELGDLERHRAQQLMKDISSNQRSRSIHFDEDFIEKLVN<br>HPNVPELVRTSPLTQREWQVLGLIYSGFSNEQIAQELDVAGTTIKTHIRNLYQKLNIANRKEAIVT<br>AENLLQLMGY	
VPA1729	MAWGQIAVVSPLVNIPVNTIEQSLLRQLPGCWGCKDKDSVFRYVNQEYAELLGHASPEECIGKT<br>DFEMSSPTTECAQEFQRQDKHVIETGESLKILDIHPYPDGRWRAHIFTKTPWRDEQGNTLGTIFY<br>GRELTDTAVIEVGYWVCRAIGTDMNHQSIFRFSNLNPKPEKLTCREQETLFLLLYGKKPQFISQV<br>MGISTKTVEGHVARLRNKFEANSKNELIDKAMEAGYGSVVPKTLLKHQLSVVLNGER	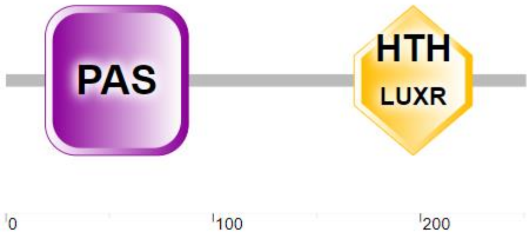

Supplemental Table S4. Differentially expressed proteins in the Δ*robA* strain versus WT strain

Gene	New locus_id	log2 fold change	*p*-value	*q*-value	Function or description
VP0005	VP_RS00030	2.797028	2.93E-10	5.41E-08	50S ribosomal protein L34 && -
VP0024	VP_RS00105	1.431259	0.000663	0.010715	hypothetical protein && -
VP0076	VP_RS00395	1.582778	0.000172	0.003701	universal stress protein UspA && PF00582:Universal stress protein family
VP0078	VP_RS00405	1.325537	0.002204	0.024835	universal stress protein UspB && PF10625:Universal stress protein B (UspB)
VP0081	VP_RS00420	2.536309	3.18E-07	2.36E-05	BON domain-containing protein && PF04972:BON domain
VP0083	VP_RS00430	4.116353	0.00137	0.018049	hypothetical protein && -
VP0087	VP_RS00450	2.63454	3.18E-05	0.001035	PA2169 family four-helix-bundle protein && PF09537:Domain of unknown function (DUF2383)
--	VP_RS00455	2.316002	3.18E-05	0.001035	hypothetical protein && -
VP0136	VP_RS00670	1.662136	0.00011	0.002755	type II secretion system major pseudopilin GspG && PF13544:Type IV pilin N-term methylation site GFxxxE|PF08334:Type II secretion system (T2SS), protein G
VP0163	VP_RS00805	1.58822	0.00205	0.023705	energy transducer TonB && PF03544:Gram-negative bacterial TonB protein C-terminal
VP0185	VP_RS00905	1.315345	0.001843	0.022419	50S ribosomal protein L28 && -
VP0218	VP_RS01070	1.525285	0.000278	0.005399	hypothetical protein && -
VP0225	VP_RS01105	1.952432	1.38E-05	0.00054	capsular polysaccharide biosynthesis protein CapF && -
VP0228	VP_RS01120	1.317656	0.004546	0.042557	oligosaccharide repeat unit polymerase && -
VP0229	VP_RS01125	1.337458	0.002002	0.023515	dTDP-4-dehydrorhamnose 3%2C5-epimerase && PF00908:dTDP-4-dehydrorhamnose 3,5-epimerase
VP0230	VP_RS01130	1.542694	0.000321	0.006097	glycosyltransferase family 4 protein && PF00534:Glycosyl transferases group 1|PF13579:Glycosyl transferase 4-like domain
VP0242	VP_RS01190	1.872268	0.000405	0.007267	DUF3135 domain-containing protein && PF11333:Protein of unknown function (DUF3135)
VP0246	VP_RS01205	1.335116	0.002752	0.02943	cell division protein ZapB && -

VP0306	VP_RS01490	1.456072	0.001826	0.022418	peptide-methionine (S)-S-oxide reductase MsrA && PF01625:Peptide methionine sulfoxide reductase
VP0309	VP_RS01505	1.989812	1.66E-05	0.000633	gamma-glutamylcyclotransferase && PF06094:Gamma-glutamyl cyclotransferase, AIG2-like
VP0310	VP_RS01510	1.487109	0.000709	0.011292	DUF2799 domain-containing protein && PF10973:Protein of unknown function (DUF2799)
VP0379	VP_RS01825	1.395127	0.005075	0.045396	ABC transporter substrate-binding protein && PF03180:NLPA lipoprotein
VP0383	VP_RS01845	1.548384	0.005794	0.049815	hypothetical protein && PF13338:Transcriptional regulator, AbiEi antitoxin, Type IV TA system
VP0394	VP_RS01885	1.643796	0.000116	0.002834	site-specific DNA-methyltransferase && PF01555:DNA methylase
VP0401	VP_RS01910	1.374615	0.001748	0.021748	hypothetical protein && -
VP0472	VP_RS02255	2.78743	0.000211	0.004399	hypothetical protein && -
VP0473	VP_RS02260	1.436125	0.003143	0.032507	hypothetical protein && -
VP0501	VP_RS02390	1.316332	0.00177	0.021888	cation-binding protein && PF01814:Hemerythrin HHE cation binding domain
VP0502	VP_RS02395	1.506699	0.000453	0.00793	DUF3545 family protein && -
VP0515	VP_RS02460	6.844554	1.65E-37	7.31E-34	hypothetical protein && -
VP0542	VP_RS02580	1.968292	5.78E-05	0.001673	hypothetical protein && -
VP0585	VP_RS02815	1.228199	0.003308	0.033672	CBS domain-containing protein && PF00571:CBS domain
VP0589	VP_RS02835	1.268909	0.003687	0.036201	preprotein translocase subunit YajC && PF02699:Preprotein translocase subunit
VP0625	VP_RS03005	1.420163	0.003616	0.035725	hypothetical protein && -
VP0712	VP_RS03415	1.258121	0.002905	0.030482	hypothetical protein && -
VP0718	VP_RS03445	2.404383	7.13E-08	6.58E-06	YbeD family protein && -
VP0728	VP_RS03495	1.99674	2.86E-06	0.000138	zinc ribbon-containing protein && PF07295:Zinc-ribbon containing domain
VP0771	VP_RS03800	1.28899	0.002981	0.031145	flagellar biosynthesis anti-sigma factor FlgM && PF04316:Anti-sigma-28 factor, FlgM

VP0777	VP_RS03830	1.318911	0.002088	0.023949	flagellar hook assembly protein FlgD && PF13861:FlgD Tudor-like domain|PF03963:Flagellar hook capping protein - N-terminal region|PF13860:FlgD Ig-like domain
VP0843	VP_RS04110	1.177745	0.004899	0.044452	succinate dehydrogenase cytochrome b556 subunit && PF01127:Succinate dehydrogenase/Fumarate reductase transmembrane subunit
VP0867	VP_RS04215	3.895729	0.003346	0.033905	hypothetical protein && -
VP0910	VP_RS04430	2.334003	5.67E-07	3.86E-05	TRAP transporter substrate-binding protein && PF03480:Bacterial extracellular solute-binding protein, family 7
VP0911	VP_RS04435	2.306014	0.000415	0.00741	TRAP transporter small permease && PF04290:Tripartite ATP-independent periplasmic transporters, DctQ component
VP0912	VP_RS04440	2.517774	1.75E-06	9.71E-05	TRAP transporter large permease && PF06808:Tripartite ATP-independent periplasmic transporter, DctM component
VP0920	VP_RS04485	1.184075	0.004693	0.043386	HU family DNA-binding protein && -
VP0952	VP_RS04665	1.316493	0.00188	0.022538	hypothetical protein && -
VP0953	VP_RS04670	1.613098	0.00026	0.005226	GGDEF domain-containing protein && PF00990:Diguanylate cyclase, GGDEF domain|PF00497:Bacterial extracellular solute-binding proteins, family 3
VP0958	VP_RS04695	2.365035	1.81E-07	1.48E-05	CBS domain-containing protein && PF00571:CBS domain
VP0985	VP_RS04820	1.350986	0.001927	0.022932	SpoVR family protein && PF04293:SpoVR like protein
VP0986	VP_RS04825	1.490931	0.000657	0.010658	YeaH/YhbH family protein && PF04285:Protein of unknown function (DUF444)
VP0988	VP_RS04830	1.356449	0.001314	0.017469	PrkA family serine protein kinase && PF06798:PrkA serine protein kinase C-terminal domain|PF08298:PrkA AAA domain
VP0990	VP_RS04835	1.561476	0.000264	0.005299	YfbU family protein && PF03887:YfbU domain
VP1012	VP_RS04940	1.498142	0.000606	0.009979	cold shock domain-containing protein CspD && -
VP1013	VP_RS04945	1.912165	1.32E-05	0.000523	ATP-dependent Clp protease adapter ClpS && PF02617:ATP-dependent Clp protease adaptor protein ClpS
VP1021	VP_RS04985	2.354921	1.37E-06	8.23E-05	YciN family protein && -

VP1076	VP_RS05245	1.210952	0.003622	0.035725	helix-turn-helix domain-containing protein && PF13730:Helix-turn-helix domain
--	VP_RS05265	1.579805	0.001402	0.018413	hypothetical protein && -
VP1125	VP_RS05475	2.057506	0.005286	0.046204	periplasmic protein && PF16036:Chalcone isomerase-like
VP1198	VP_RS05820	1.649126	0.001091	0.015431	(Fe-S)-binding protein && PF02754:Cysteine-rich domain
VP1241	VP_RS06025	2.127283	6.85E-07	4.59E-05	TfoX/Sxy family DNA transformation protein && PF04993:TfoX N-terminal domain|PF04994:TfoX C-terminal domain
VP1245	VP_RS06040	1.344944	0.001636	0.020692	cyclic nucleotide-binding domain-containing protein && PF00512:His Kinase A (phospho-acceptor) domain|PF00027:Cyclic nucleotide-binding domain|PF02518:Histidine kinase-, DNA gyrase B-, and HSP90-like ATPase
VP1261	VP_RS06120	1.757697	0.000354	0.006532	hypothetical protein && -
VP1265	VP_RS06140	1.345606	0.003527	0.035021	hypothetical protein && -
VP1318	VP_RS06400	2.165218	0.001436	0.018648	hypothetical protein && -
VP1319	VP_RS06405	2.510587	3.18E-05	0.001035	polysaccharide pyruvyl transferase family protein && PF04230:Polysaccharide pyruvyl transferase
VP1320	VP_RS06410	2.825346	1.52E-06	8.87E-05	2-C-methyl-D-erythritol 4-phosphate cytidylyltransferase && PF01128:2-C-methyl-D-erythritol 4-phosphate cytidylyltransferase
VP1321	VP_RS06415	4.376648	2.21E-09	2.79E-07	NAD-dependent epimerase/dehydratase family protein && PF01370:NAD dependent epimerase/dehydratase family
VP1361	VP_RS06615	1.944967	0.00124	0.016682	MlaE family lipid ABC transporter permease subunit && PF13466:STAS domain|PF02405:Permease MlaE
VP1363	VP_RS06620	3.09329	0.000155	0.003477	ATP-binding cassette domain-containing protein && PF00005:ABC transporter
VP1374	VP_RS06685	1.372166	0.001516	0.019572	SLC13 family permease && PF00939:Sodium:sulfate symporter transmembrane region
VP1375	VP_RS06690	1.48811	0.000892	0.013483	cyclic nucleotide-binding domain-containing protein && PF02518:Histidine kinase-, DNA gyrase B-, and HSP90-like ATPase|PF00027:Cyclic nucleotide-binding domain

VP1408	VP_RS06840	1.335832	0.003738	0.036537	type VI secretion system membrane subunit TssM && PF06744:Type VI secretion protein IcmF C-terminal|PF14331:ImcF-related N-terminal domain|PF06761:Intracellular multiplication and human macrophage-killing
VP1425	VP_RS06920	1.591448	0.001585	0.020278	iron-containing alcohol dehydrogenase && PF00465:Iron-containing alcohol dehydrogenase
VP1427	VP_RS06930	1.359376	0.001305	0.0174	c-di-GMP phosphodiesterase && PF13487:HD domain
VP1431	VP_RS06945	1.425505	0.002201	0.024835	ATP-binding cassette domain-containing protein && PF00005:ABC transporter|PF00664:ABC transporter transmembrane region
VP1432	VP_RS06950	1.60968	0.002684	0.028991	ATP-binding cassette domain-containing protein && PF00664:ABC transporter transmembrane region|PF00005:ABC transporter
VP1434	VP_RS06955	2.901956	2.96E-06	0.000141	type II secretion system protein && -
VP1455	VP_RS07060	1.304528	0.003396	0.034069	outer membrane beta-barrel protein && PF13505:Outer membrane protein beta-barrel domain
VP1473	VP_RS07135	2.772851	0.001174	0.016044	CpsD/CapB family tyrosine-protein kinase && PF01656:CobQ/CobB/MinD/ParA nucleotide binding domain
VP1475	VP_RS07145	1.786715	0.001341	0.017732	OmpA family protein && PF00691:OmpA family
VP1490	VP_RS07225	1.951916	0.005291	0.046204	hypothetical protein && -
VP1508	VP_RS07300	2.418189	2.01E-06	0.000106	DUF3305 domain-containing protein && PF11749:Protein of unknown function (DUF3305)
VP1517	VP_RS07345	1.287195	0.002692	0.029004	type IV secretion protein Rhs && PF14414:A nuclease of the HNH/ENDO VII superfamily with conserved WHH|PF03527:RHS protein
VP1518	VP_RS07350	1.642087	0.000175	0.003726	SMI1/KNR4 family protein && -
VP1545	VP_RS07475	1.689638	0.002863	0.030257	hypothetical protein && -
VP1554	VP_RS07515	2.484475	1.75E-05	0.00065	hypothetical protein && -
VP1589	VP_RS07660	1.420886	0.000935	0.013929	macrodomain Ter protein MatP && PF06303:Organiser of macrodomain of Terminus of chromosome

VP1599	VP_RS07700	1.419163	0.001961	0.023221	cell division protein ZapC && PF07126:Cell-division protein ZapC
VP1627	VP_RS07830	1.452796	0.001108	0.015532	acylphosphatase && -
VP1706	VP_RS08215	1.716925	4.86E-05	0.001466	hypothetical protein && -
--	VP_RS08250	1.793236	2.93E-05	0.000997	hypothetical protein && -
VP1722	VP_RS08295	2.174562	0.000337	0.006319	diaminobutyrate acetyltransferase && PF00583:Acetyltransferase (GNAT) family
VP1749	VP_RS08415	1.448765	0.000937	0.013929	hypothetical protein && -
--	VP_RS08455	1.51549	0.001406	0.018413	sugar O-acetyltransferase && PF12464:Maltose acetyltransferase|PF14602:Hexapeptide repeat of succinyl-transferase
--	VP_RS08460	3.075909	3.62E-10	5.78E-08	hypothetical protein && -
VP1881	VP_RS09160	4.04084	1.29E-15	6.37E-13	EAL domain-containing protein && PF00563:EAL domain
VP1894	VP_RS09210	2.405614	0.002142	0.024262	hypothetical protein && -
VP1897	VP_RS09220	2.139266	4.85E-06	0.000219	hypothetical protein && -
VP1908	VP_RS09270	1.963655	2.71E-05	0.000943	sensor histidine kinase && PF08269:Cache domain|PF07730:Histidine kinase|PF02518:Histidine kinase-, DNA gyrase B-, and HSP90-like ATPase
VP1911	VP_RS09285	1.654695	0.002073	0.023845	TRAP transporter substrate-binding protein && PF03480:Bacterial extracellular solute-binding protein, family 7
VP1916	VP_RS09310	1.656165	0.000273	0.005354	L%2CD-transpeptidase family protein && PF03734:L,D-transpeptidase catalytic domain|PF01471:Putative peptidoglycan binding domain
VP1917	VP_RS09315	1.971127	7.79E-05	0.002106	DUF1513 domain-containing protein && PF07433:Protein of unknown function (DUF1513)
VP1918	VP_RS09320	1.647282	0.000856	0.013071	iron-regulated protein A && PF09375:Imelysin
VP1919	VP_RS09325	1.47934	0.002411	0.026425	c-type cytochrome && PF06537:Di-haem oxidoreductase, putative peroxidase
VP1920	VP_RS09330	1.50052	0.000917	0.013763	peptidase && PF09375:Imelysin
VP1928	VP_RS09365	1.942661	1.03E-05	0.000429	cytochrome c nitrite reductase pentaheme subunit && PF13435:Cytochrome c554 and c-prime
VP1939	VP_RS09420	1.194718	0.004382	0.041285	transcriptional regulator && PF13740:ACT domain

VP1952	VP_RS09500	2.105709	3.75E-05	0.001211	calcium-binding protein && -
VP1962	VP_RS09550	1.369767	0.005121	0.045655	Crp/Fnr family transcriptional regulator && PF00027:Cyclic nucleotide-binding domain|PF13545:Crp-like helix-turn-helix domain
VP1977	VP_RS09610	2.916608	9.60E-11	2.12E-08	DUF4250 domain-containing protein && -
VP1979	VP_RS09620	2.325379	1.23E-07	1.07E-05	EAL domain-containing protein && PF00563:EAL domain|PF00989:PAS fold
VP2044	VP_RS09940	1.491926	0.000587	0.009701	hypothetical protein && -
VP2120	VP_RS10305	1.264181	0.004489	0.042111	SDR family oxidoreductase && PF00106:short chain dehydrogenase
VP2127	VP_RS10340	1.424968	0.001432	0.018648	Hpt domain-containing protein && PF01627:Hpt domain
VP2129	VP_RS10350	2.459554	2.60E-05	0.000914	YejL family protein && -
VP2147	VP_RS10435	1.820986	5.35E-05	0.001589	hypothetical protein && -
VP2167	VP_RS10525	1.864004	0.000471	0.008052	antibiotic biosynthesis monooxygenase && -
VP2170	VP_RS10540	1.656249	0.000297	0.005739	GlsB/YeaQ/YmgE family stress response membrane protein && -
VP2190	VP_RS10635	1.808908	7.85E-05	0.002106	tRNA pseudouridine(38-40) synthase TruA && PF01416:tRNA pseudouridine synthase
VP2207	VP_RS10710	2.272432	1.78E-07	1.48E-05	hypothetical protein && -
VP2280	VP_RS11065	1.257436	0.004177	0.039945	NAD(P)H:quinone oxidoreductase && PF03358:NADPH-dependent FMN reductase
VP2299	VP_RS11160	1.739881	5.43E-05	0.001597	nitrogen regulatory protein P-II && PF00543:Nitrogen regulatory protein P-II
VP2362	VP_RS11470	1.786677	2.52E-05	0.000894	outer membrane protein OmpK && PF03502:Nucleoside-specific channel-forming protein, Tsx
VP2379	VP_RS11555	1.338792	0.002037	0.023705	YebG family protein && -
VP2436	VP_RS11815	1.183523	0.004755	0.043596	deoxyribose-phosphate aldolase && PF01791:DeoC/LacD family aldolase
VP2453	VP_RS11900	1.16335	0.005301	0.046204	30S ribosomal protein S15 && -
VP2499	VP_RS12135	1.445125	0.001147	0.015865	DNA/RNA nuclease SfsA && PF03749:Sugar fermentation stimulation protein
VP2500	VP_RS12140	2.378606	5.06E-08	4.98E-06	RNA polymerase-binding protein DksA && PF01258:Prokaryotic dksA/traR C4-type zinc finger
VP2516	VP_RS12200	1.529973	0.000324	0.006112	TetR/AcrR family transcriptional regulator && PF00440:Bacterial regulatory proteins, tetR family

VP2545	VP_RS12345	1.942661	1.03E-05	0.000429	oxaloacetate decarboxylase subunit gamma && -
VP2547	VP_RS12400	1.511153	0.000395	0.007154	aspartate kinase && PF00696:Amino acid kinase family
VP2628	VP_RS12785	1.548384	0.005794	0.049815	membrane-bound lytic murein transglycosylase MltC && PF11873:Domain of unknown function (DUF3393)|PF01464:Transglycosylase SLT domain
VP2683	VP_RS13160	2.406258	4.01E-08	4.03E-06	PhoH family protein && PF13638:PIN domain|PF02562:PhoH-like protein
VP2697	VP_RS13230	1.620106	0.00183	0.022418	hypothetical protein && -
VP2699	VP_RS13240	1.893343	2.79E-05	0.000957	MSHA biogenesis protein MshF && -
VP2703	VP_RS13260	1.29381	0.004271	0.040699	AAA family ATPase && PF13401:AAA domain
VP2723	VP_RS13380	1.64054	0.001621	0.020566	hypothetical protein && -
VP2759	VP_RS13545	3.527621	1.64E-06	9.43E-05	N-acetyl-gamma-glutamyl-phosphate reductase && PF01118:Semialdehyde dehydrogenase, NAD binding domain|PF02774:Semialdehyde dehydrogenase, dimerisation domain
VP2791	VP_RS13695	2.030547	0.000974	0.014238	YheU family protein && -
VP2803	VP_RS13740	2.150332	0.001019	0.014562	hypothetical protein && -
VP2868	VP_RS14110	2.581744	0.000884	0.013405	DUF4212 domain-containing protein && -
VP2878	VP_RS14155	1.507125	0.000419	0.007448	acetate--CoA ligase && PF13193:AMP-binding enzyme C-terminal domain|PF00501:AMP-binding enzyme|PF16177:Acetyl-coenzyme A synthetase N-terminus
VP2907	VP_RS14340	1.696589	0.000344	0.006423	hypothetical protein && -
VP2935	VP_RS14535	1.576985	0.00022	0.004532	RNA-binding protein && PF00076:RNA recognition motif. (a.k.a. RRM, RBD, or RNP domain)
VP2964	VP_RS14670	1.853025	1.68E-05	0.000635	hypothetical protein && -
VP2965	VP_RS14675	2.781575	0.000466	0.008052	DUF2500 domain-containing protein && PF10694:Protein of unknown function (DUF2500)
VP2973	VP_RS14715	1.530016	0.003323	0.033744	homoserine/homoserine lactone efflux protein && PF01810:LysE type translocator
VP2989	VP_RS14795	1.401264	0.001224	0.01653	uroporphyrinogen-III synthase && PF02602:Uroporphyrinogen-III synthase HemD

VP2996	VP_RS14845	1.763087	0.001861	0.022456	2Fe-2S iron-sulfur cluster binding domain-containing protein && -
VP3057	VP_RS15160	2.699565	2.17E-07	1.72E-05	YifB family Mg chelatase-like AAA ATPase && PF01078:Magnesium chelatase, subunit ChlI|PF13335:Magnesium chelatase, subunit ChlI C-terminal|PF13541:Subunit ChlI of Mg-chelatase
VP3073	VP_RS15240	1.384988	0.000982	0.014259	F0F1 ATP synthase subunit B && PF00430:ATP synthase B/B' CF(0)
VP3074	VP_RS15245	1.534016	0.000324	0.006112	F0F1 ATP synthase subunit C && -
VP3075	VP_RS15250	1.596984	0.000154	0.003477	F0F1 ATP synthase subunit A && PF00119:ATP synthase A chain
VP3076	VP_RS15255	1.728295	4.46E-05	0.001392	F0F1 ATP synthase subunit I && PF03899:ATP synthase I chain
VPA0002	VP_RS15285	2.144687	0.000974	0.014238	hypothetical protein && -
VPA0022	VP_RS15370	1.977313	0.000853	0.013071	DUF2268 domain-containing protein && PF10026:Predicted Zn-dependent protease (DUF2268)
VPA0039	VP_RS15435	2.093446	2.16E-06	0.000109	DUF3081 domain-containing protein && -
--	VP_RS15460	1.699741	0.000134	0.003129	hypothetical protein && -
VPA0051	VP_RS15490	1.378777	0.001702	0.021348	sodium:proton antiporter && PF03600:Citrate transporter
VPA0058	VP_RS15525	1.819694	3.83E-05	0.001228	hypothetical protein && -
VPA0101	VP_RS15735	1.343567	0.003201	0.032884	hypothetical protein && -
VPA0150	VP_RS16005	1.870219	2.52E-05	0.000894	TonB-dependent siderophore enterobactin receptor PeuA && PF00593:TonB dependent receptor|PF07715:TonB-dependent Receptor Plug Domain
VPA0151	VP_RS16010	1.670339	0.001299	0.01738	DUF3450 domain-containing protein && PF11932:Protein of unknown function (DUF3450)
VPA0155	VP_RS16030	1.627072	0.002777	0.029629	energy transducer TonB && PF03544:Gram-negative bacterial TonB protein C-terminal
VPA0178	VP_RS16140	1.202317	0.004586	0.042748	YggL family protein && PF04320:Protein with unknown function (DUF469)
VPA0210	VP_RS16285	2.664437	7.49E-07	4.95E-05	hypothetical protein && -
VPA0248	VP_RS16465	2.322523	8.61E-08	7.78E-06	OmpA family protein && PF00691:OmpA family|PF01389:OmpA-like transmembrane domain

VPA0263	VP_RS16535	1.491869	0.003233	0.033066	flagellar basal body P-ring formation protein FlgA && PF13144:Chaperone for flagella basal body P-ring formation
VPA0320	VP_RS16805	1.679547	7.65E-05	0.002105	copper resistance protein NlpE && PF04170:NlpE N-terminal domain
VPA0329	VP_RS16840	1.708124	0.00069	0.011066	L-Ala-D/L-Glu epimerase && PF13378:Enolase C-terminal domain-like|PF02746:Mandelate racemase / muconate lactonizing enzyme, N-terminal domain
VPA0347	VP_RS16905	1.267943	0.003632	0.035742	hypothetical protein && -
VPA0358	VP_RS16960	4.508586	1.36E-11	3.18E-09	helix-turn-helix transcriptional regulator && PF00196:Bacterial regulatory proteins, luxR family
VPA0359	VP_RS16965	3.171025	0.000349	0.006484	helix-turn-helix transcriptional regulator && PF12844:Helix-turn-helix domain
VPA0360	VP_RS16970	4.459394	1.06E-08	1.15E-06	GGDEF domain-containing protein && PF00990:Diguanylate cyclase, GGDEF domain
VPA0371	VP_RS17020	1.403286	0.001576	0.020225	hypothetical protein && -
VPA0420	VP_RS17245	1.340519	0.002021	0.023658	TetR/AcrR family transcriptional regulator && -
--	VP_RS17345	1.732775	0.000277	0.005394	His-Xaa-Ser system protein HxsD && -
VPA0445	VP_RS17365	1.517475	0.005225	0.046204	TIGR03982 family His-Xaa-Ser system protein && -
VPA0447	VP_RS17375	1.897975	2.43E-05	0.000875	hypothetical protein && PF01734:Patatin-like phospholipase
VPA0448	VP_RS17380	1.607543	0.000553	0.009163	hypothetical protein && -
VPA0457	VP_RS17420	3.149644	0.000112	0.002764	hypothetical protein && -
VPA0458	VP_RS17425	1.889154	0.000241	0.004908	hypothetical protein && -
VPA0460	VP_RS17435	1.322847	0.001883	0.022538	hypothetical protein && -
VPA0463	VP_RS17450	3.137692	1.04E-10	2.19E-08	hypothetical protein && -
VPA0476	VP_RS17510	1.59421	0.000615	0.010087	sensor domain-containing diguanylate cyclase && PF01590:GAF domain|PF00990:Diguanylate cyclase, GGDEF domain
VPA0515	VP_RS17695	1.945429	0.00015	0.003455	GFA family protein && PF04828:Glutathione-dependent formaldehyde-activating enzyme
VPA0518	VP_RS17710	1.628816	0.000235	0.004819	phosphodiesterase && PF00990:Diguanylate cyclase, GGDEF domain|PF00563:EAL domain

VPA0527	VP_RS17745	2.545164	8.71E-09	9.87E-07	porin && PF13609:Gram-negative porin
VPA0552	VP_RS17860	1.650299	8.91E-05	0.00228	cold-shock protein && -
VPA0557	VP_RS17880	1.425744	0.003431	0.034291	outer membrane lipoprotein-sorting protein && -
VPA0570	VP_RS17940	1.536413	0.000873	0.013277	hypothetical protein && -
--	VP_RS17945	2.658861	3.20E-07	2.36E-05	hypothetical protein && -
VPA0588	VP_RS18030	2.823213	3.65E-10	5.78E-08	hypothetical protein && -
VPA0606	VP_RS18115	1.309582	0.004332	0.041073	helix-turn-helix transcriptional regulator && PF12833:Helix-turn-helix domain
VPA0622	VP_RS18190	2.30791	0.001185	0.016044	acyl-CoA dehydrogenase && PF02771:Acyl-CoA dehydrogenase, N-terminal domain|PF02770:Acyl-CoA dehydrogenase, middle domain|PF00441:Acyl-CoA dehydrogenase, C-terminal domain
VPA0657	VP_RS18345	1.754592	0.000469	0.008052	siderophore ABC transporter substrate-binding protein && PF01497:Periplasmic binding protein
VPA0658	VP_RS18350	2.208427	0.000273	0.005354	ABC transporter permease && PF01032:FecCD transport family
VPA0666	VP_RS18385	1.608027	0.000318	0.006064	membrane protein && -
VPA0668	VP_RS18390	1.403991	0.000845	0.013029	retention module-containing protein && -
VPA0670	VP_RS18400	1.621076	0.000167	0.003631	hypothetical protein && -
VPA0676	VP_RS18430	2.373709	1.18E-05	0.000481	hypothetical protein && -
VPA0680	VP_RS18450	1.451322	0.002982	0.031145	arylsulfatase && PF14707:C-terminal region of aryl-sulfatase|PF00884:Sulfatase
VPA0703	VP_RS18555	1.957704	6.00E-06	0.000266	hypothetical protein && -
VPA0766	VP_RS18845	2.11422	2.85E-06	0.000138	YbaK/EbsC family protein && PF04073:Aminoacyl-tRNA editing domain
VPA0844	VP_RS19205	2.765349	3.66E-10	5.78E-08	DUF479 domain-containing protein && PF04336:Protein of unknown function, DUF479
VPA0853	VP_RS19250	2.615823	5.34E-09	6.22E-07	DUF1097 domain-containing protein && PF06496:Protein of unknown function (DUF1097)
VPA0910	VP_RS19530	1.593483	0.00206	0.023751	helix-turn-helix transcriptional regulator && -
VPA0912	VP_RS19545	2.791632	4.11E-05	0.00129	LysR family transcriptional regulator && PF03466:LysR substrate binding domain|PF00126:Bacterial regulatory helix-turn-helix protein, lysR family

VPA0914	VP_RS19550	1.589142	0.000446	0.007845	hypothetical protein && -
VPA0915	VP_RS19555	1.829642	0.000123	0.002951	universal stress protein && PF00582:Universal stress protein family
VPA0970	VP_RS19810	2.026379	0.001866	0.022456	DUF1501 domain-containing protein && PF07394:Protein of unknown function (DUF1501)
VPA0981	VP_RS19865	1.665282	0.000158	0.003512	anaerobic C4-dicarboxylate transporter && PF03605:Anaerobic c4-dicarboxylate membrane transporter
VPA0983	VP_RS19875	1.984684	0.002322	0.025853	DUF4174 domain-containing protein && PF13778:Domain of unknown function (DUF4174)
VPA1018	VP_RS20055	2.334039	8.48E-06	0.000365	hypothetical protein && PF08212:Lipocalin-like domain
VPA1024	VP_RS20085	1.613084	0.001835	0.022418	hypothetical protein && -
VPA1025	VP_RS20090	2.175314	0.001999	0.023515	type VI secretion protein && -
VPA1027	VP_RS20100	1.330311	0.0021	0.024032	type VI secretion system tube protein Hcp && PF05638:Type VI secretion system effector, Hcp
VPA1036	VP_RS20145	1.532239	0.00161	0.02052	type VI secretion system protein TssA && PF06812:ImpA, N-terminal, type VI secretion system
VPA1037	VP_RS20150	1.403607	0.005712	0.049306	serine/threonine-protein phosphatase && PF13672:Protein phosphatase 2C
VPA1038	VP_RS20155	1.971127	7.79E-05	0.002106	type VI secretion system-associated protein TagF && PF09867:Uncharacterized protein conserved in bacteria (DUF2094)
VPA1041	VP_RS20170	1.69663	0.000252	0.005104	type VI secretion system baseplate subunit TssK && PF05936:Bacterial Type VI secretion, VC_A0110, EvfL, ImpJ, VasE
VPA1045	VP_RS20190	1.351643	0.003891	0.037781	response regulator && PF00072:Response regulator receiver domain
VPA1046	VP_RS20195	3.032827	2.06E-06	0.000107	hypothetical protein && -
--	VP_RS20345	2.188858	3.95E-05	0.001259	hypothetical protein && -
VPA1096	VP_RS20425	1.695725	8.64E-05	0.002236	DUF4136 domain-containing protein && PF13590:Domain of unknown function (DUF4136)
VPA1144	VP_RS20650	3.307852	0.000167	0.003631	c-type cytochrome && PF00034:Cytochrome c

VPA1149	VP_RS20675	1.699104	0.003145	0.032507	ABC transporter substrate-binding protein && PF13458:Periplasmic binding protein
VPA1197	VP_RS20900	2.630373	1.74E-06	9.71E-05	ferredoxin-type protein NapF && PF12838:4Fe-4S dicluster domain
VPA1216	VP_RS21000	1.233407	0.005519	0.047823	flagellar brake protein && PF12945:Flagellar protein YcgR|PF07238:PilZ domain
VPA1226	VP_RS21045	1.17643	0.005273	0.046204	DUF496 family protein && PF04363:Protein of unknown function (DUF496)
VPA1250	VP_RS21150	1.999096	8.45E-06	0.000365	3%2C4-dihydroxy-2-butanone-4-phosphate synthase && PF00926:3,4-dihydroxy-2-butanone 4-phosphate synthase
--	VP_RS21225	2.274986	0.005286	0.046204	bacteriocin immunity protein && -
--	VP_RS21440	1.721896	8.27E-05	0.002154	hypothetical protein && -
VPA1343	VP_RS21600	2.029524	2.07E-05	0.000752	hypothetical protein && -
VPA1345	VP_RS21605	2.095187	1.96E-06	0.000106	hypothetical protein && -
VPA1350	VP_RS21630	1.603028	0.000546	0.009117	VPA1350 family putative T3SS effector && -
--	VP_RS21750	1.343567	0.003201	0.032884	IS5 family transposase && PF13737:Transposase DDE domain
VPA1390	VP_RS21805	2.145658	0.000125	0.002969	type I-F CRISPR-associated endoribonuclease Cas6/Csy4 && PF09618:CRISPR-associated protein (Cas_Csy4)
VPA1394	VP_RS21825	1.28007	0.004456	0.041887	AAA family ATPase && PF13401:AAA domain
VPA1403	VP_RS21870	3.719491	2.64E-13	6.87E-11	undecaprenyl-phosphate glucose phosphotransferase && PF13727:CoA-binding domain|PF02397:Bacterial sugar transferase
VPA1404	VP_RS21875	4.235381	2.85E-16	2.10E-13	outer membrane beta-barrel protein && PF10082:Putative beta-barrel porin 2
VPA1405	VP_RS21880	4.502775	1.11E-13	3.50E-11	polysaccharide export protein && PF02563:Polysaccharide biosynthesis/export protein|PF10531:SLBB domain
--	VP_RS21885	6.229483	7.12E-26	1.58E-22	polysaccharide biosynthesis tyrosine autokinase && PF13807:G-rich domain on putative tyrosine kinase|PF02706:Chain length determinant protein
VPA1407	VP_RS21890	4.998852	1.18E-15	6.37E-13	protein CpsE && PF10364:Putative capsular polysaccharide synthesis protein
VPA1408	VP_RS21895	4.802735	2.09E-17	2.31E-14	glycosyltransferase && PF00534:Glycosyl transferases group 1|PF13439:Glycosyltransferase Family 4
VPA1409	VP_RS21900	4.393499	1.96E-14	7.24E-12	O-antigen ligase family protein && PF13425:O-antigen ligase like membrane protein

VPA1410	VP_RS21905	4.387875	8.20E-15	3.30E-12	capsular polysaccharide biosynthesis protein && PF10364:Putative capsular polysaccharide synthesis protein
VPA1411	VP_RS21910	4.986131	4.00E-18	5.91E-15	glycosyltransferase && PF13579:Glycosyl transferase 4-like domain|PF00534:Glycosyl transferases group 1
VPA1412	VP_RS21915	4.20582	1.59E-13	4.68E-11	oligosaccharide flippase family protein && PF01943:Polysaccharide biosynthesis protein|PF14667:Polysaccharide biosynthesis C-terminal domain
VPA1413	VP_RS21920	4.828401	2.40E-06	0.00012	hypothetical protein && PF04892:VanZ like family
VPA1441	VP_RS22060	1.642038	0.000707	0.011292	hypothetical protein && -
VPA1443	VP_RS22070	2.92254	1.55E-09	2.01E-07	HlyD family type I secretion periplasmic adaptor subunit && PF13533:Biotin-lipoyl like|PF13437:HlyD family secretion protein
VPA1444	VP_RS22075	2.508845	4.24E-07	3.03E-05	type I secretion system permease/ATPase && PF00005:ABC transporter
VPA1445	VP_RS22080	3.319453	8.92E-09	9.87E-07	calcium-binding protein && -
VPA1446	VP_RS22085	2.903959	3.13E-09	3.85E-07	helix-turn-helix transcriptional regulator && PF00196:Bacterial regulatory proteins, luxR family
VPA1447	VP_RS22090	3.762673	5.45E-12	1.34E-09	LuxR family transcriptional regulator && PF00196:Bacterial regulatory proteins, luxR family
VPA1469	VP_RS22195	1.700656	5.53E-05	0.00161	hypothetical protein && -
VPA1479	VP_RS22240	1.58032	0.00555	0.047998	DUF3302 domain-containing protein && PF11742:Protein of unknown function (DUF3302)
VPA1487	VP_RS22280	2.712046	5.05E-09	6.04E-07	GNAT family N-acetyltransferase && PF00583:Acetyltransferase (GNAT) family
VPA1500	VP_RS22340	1.330014	0.001838	0.022418	helix-turn-helix transcriptional regulator && -
VPA1504	VP_RS22360	1.931427	0.001107	0.015532	fimbrial biogenesis outer membrane usher protein && PF00577:Outer membrane usher protein
VPA1531	VP_RS22480	1.932135	0.000111	0.002761	serine protease && PF00089:Trypsin
VPA1578	VP_RS22705	1.887148	0.000158	0.003512	fusaric acid resistance family protein && PF04632:Fusaric acid resistance protein family
VPA1596	VP_RS22795	1.51182	0.003832	0.037296	GNAT family N-acetyltransferase && PF00583:Acetyltransferase (GNAT) family

VPA1600	VP_RS22815	1.720215	0.002232	0.02502	MarC family protein && PF01914:MarC family integral membrane protein
VPA1601	VP_RS22820	1.6023	0.000396	0.007154	oxygen-insensitive NADPH nitroreductase && PF00881:Nitroreductase family
VPA1612	VP_RS22875	1.493981	0.001027	0.014626	membrane protein && PF07670:Nucleoside recognition
VPA1628	VP_RS22945	1.823618	0.000113	0.002785	PTS transporter subunit EIIB && -
VPA1629	VP_RS22950	2.410959	5.18E-08	4.99E-06	ribosome small subunit-dependent GTPase A && PF03193:Protein of unknown function, DUF258
--	VP_RS23140	1.88641	0.002125	0.024256	DUF302 domain-containing protein && PF03625:Domain of unknown function DUF302
VPA1678	VP_RS23185	1.310185	0.004568	0.042677	arabinose operon transcriptional regulator AraC && PF12833:Helix-turn-helix domain|PF02311:AraC-like ligand binding domain
VPA1715	VP_RS23355	1.998849	3.20E-06	0.000149	ABC transporter ATP-binding protein && PF00664:ABC transporter transmembrane region|PF00005:ABC transporter
VPA1734	VP_RS23440	2.12976	5.53E-06	0.000247	sulfate adenylyltransferase && PF06035:Bacterial transglutaminase-like cysteine proteinase BTLCP
--	VP_RS23530	1.959463	0.000444	0.007841	hypothetical protein && -
--	VP_RS23540	1.846787	8.15E-05	0.002149	hypothetical protein && -
--	VP_RS23570	2.461137	0.003149	0.032507	hypothetical protein && -
--	VP_RS23635	2.169363	8.81E-07	5.57E-05	hypothetical protein && -
--	VP_RS23690	1.834213	5.23E-05	0.001565	hypothetical protein && -

VP0007	VP_RS00040	-1.98057	0.000367	0.006735	amino acid ABC transporter permease && PF00528:Binding-protein-dependent transport system inner membrane component
VP0008	VP_RS00045	-1.65434	0.000174	0.003724	amino acid ABC transporter substrate-binding protein && PF00497:Bacterial extracellular solute-binding proteins, family 3
VP0018	VP_RS00075	-2.84216	1.90E-10	3.65E-08	Hsp20 family protein && PF00011:Hsp20/alpha crystallin family
VP0021	VP_RS00090	-1.44518	0.000675	0.010868	glycine--tRNA ligase subunit beta && PF02092:Glycyl-tRNA synthetase beta subunit|PF05746:DALR anticodon binding domain

VP0028	VP_RS00125	-1.44109	0.003361	0.033976	oxidoreductase && PF00107:Zinc-binding dehydrogenase
VP0038	VP_RS00200	-1.59667	0.000184	0.003885	**multidrug** efflux RND transporter permease subunit VmeD && PF00873:AcrB/AcrD/AcrF family
VP0039	VP_RS00205	-1.56326	0.000438	0.00775	multidrug efflux RND transporter periplasmic adaptor subunit VmeC && PF16576:Barrel-sandwich domain of CusB or HlyD membrane-fusion
VP0040	VP_RS00210	-1.52466	0.001424	0.018604	TetR/AcrR family transcriptional regulator && PF00440:Bacterial regulatory proteins, tetR family|PF16295:Tetracycline repressor, C-terminal all-alpha domain
VP0060	VP_RS00315	-1.53271	0.005056	0.045387	multidrug transporter subunit MdtJ && PF00893:Small Multidrug Resistance protein
VP0061	VP_RS00320	-1.93035	0.00091	0.0137	multidrug transporter && PF00893:Small Multidrug Resistance protein
VP0066	VP_RS00345	-1.83786	0.001341	0.017732	xanthosine phosphorylase && PF01048:Phosphorylase superfamily
VP0068	VP_RS00355	-1.51038	0.000477	0.008119	glutathione-disulfide reductase && PF07992:Pyridine nucleotide-disulphide oxidoreductase|PF02852:Pyridine nucleotide-disulphide oxidoreductase, dimerisation domain
VP0159	VP_RS00785	-1.6648	0.000118	0.002857	bifunctional GTP diphosphokinase/guanosine-3'%2C5'-bis pyrophosphate 3'-pyrophosphohydrolase && PF13291:ACT domain|PF13328:HD domain|PF04607:Region found in RelA / SpoT proteins|PF02824:TGS domain
VP0239	VP_RS01175	-1.74066	5.87E-05	0.001687	triose-phosphate isomerase && PF00121:Triosephosphate isomerase
VP0243	VP_RS01195	-1.3619	0.003926	0.038036	transcriptional regulator && PF13412:Winged helix-turn-helix DNA-binding
VP0249	VP_RS01220	-2.45738	2.28E-07	1.77E-05	HslU--HslV peptidase ATPase subunit && PF00004:ATPase family associated with various cellular activities (AAA)|PF07724:AAA domain (Cdc48 subfamily)
VP0250	VP_RS01225	-2.43744	8.13E-05	0.002149	ATP-dependent protease subunit HslV && PF00227:Proteasome subunit
VP0291	VP_RS01425	-2.94018	6.89E-10	9.84E-08	uroporphyrinogen-III C-methyltransferase && PF00590:Tetrapyrrole (Corrin/Porphyrin) Methylases
VP0325	VP_RS01570	-1.68455	6.91E-05	0.001936	malate dehydrogenase && PF02866:lactate/malate dehydrogenase, alpha/beta C-terminal domain|PF00056:lactate/malate dehydrogenase, NAD binding domain

VP0356	VP_RS01720	-1.79881	3.06E-05	0.001034	pyruvate kinase PykF && PF00224:Pyruvate kinase, barrel domain|PF02887:Pyruvate kinase, alpha/beta domain
VP0358	VP_RS01725	-2.0602	1.57E-05	0.000604	DeoR family transcriptional regulator && PF00455:DeoR C terminal sensor domain|PF08220:DeoR-like helix-turn-helix domain
VP0359	VP_RS01730	-2.16702	9.42E-07	5.79E-05	glutamine--fructose-6-phosphate transaminase (isomerizing) && PF13522:Glutamine amidotransferase domain|PF01380:SIS domain
VP0363	VP_RS01750	-1.30529	0.002807	0.029806	glycerol dehydrogenase && PF00465:Iron-containing alcohol dehydrogenase
VP0369	VP_RS01780	-1.61375	0.000649	0.010564	mannitol-1-phosphate 5-dehydrogenase && PF01232:Mannitol dehydrogenase Rossmann domain|PF08125:Mannitol dehydrogenase C-terminal domain
VP0370	VP_RS01785	-1.64991	0.000271	0.005354	PTS mannitol transporter subunit IICBA && PF00359:Phosphoenolpyruvate-dependent sugar phosphotransferase system, EIIA 2|PF02378:Phosphotransferase system, EIIC|PF02302:PTS system, Lactose/Cellobiose specific IIB subunit
--	VP_RS01795	-3.0255	0.00237	0.026175	hypothetical protein && -
VP0398	VP_RS01895	-1.771	0.000823	0.012737	MarR family transcriptional regulator && -
VP0408	VP_RS01950	-1.33591	0.003217	0.032969	tRNA (adenosine(37)-N6)-threonylcarbamoyltransferase complex transferase subunit TsaD && PF00814:Glycoprotease family
VP0433	VP_RS02075	-1.60997	0.000153	0.003477	Do family serine endopeptidase && PF13180:PDZ domain|PF00595:PDZ domain (Also known as DHR or GLGF)|PF13365:Trypsin-like peptidase domain
VP0561	VP_RS02670	-1.19519	0.004596	0.042757	ATP-dependent chaperone ClpB && PF07724:AAA domain (Cdc48 subfamily)|PF10431:C-terminal, D2-small domain, of ClpB protein|PF00004:ATPase family associated with various cellular activities (AAA)|PF02861:Clp amino terminal domain, pathogenicity island component
VP0567	VP_RS02730	-1.28053	0.003552	0.03519	tRNA 5-hydroxyuridine modification protein YegQ && PF16325:Peptidase family U32 C-terminal domain|PF01136:Peptidase family U32
VP0593	VP_RS02850	-1.47501	0.001138	0.015841	inositol-1-monophosphatase && PF00459:Inositol monophosphatase family

VP0615	VP_RS02955	-2.00216	1.04E-05	0.000429	exodeoxyribonuclease VII large subunit && PF02601:Exonuclease VII, large subunit|PF13742:OB-fold nucleic acid binding domain
VP0616	VP_RS02960	-2.15713	8.09E-07	5.27E-05	IMP dehydrogenase && PF00478:IMP dehydrogenase / GMP reductase domain|PF00571:CBS domain
VP0617	VP_RS02965	-1.81244	2.75E-05	0.00095	glutamine-hydrolyzing GMP synthase && PF00117:Glutamine amidotransferase class-I|PF02540:NAD synthase|PF00958:GMP synthase C terminal domain
VP0620	VP_RS02980	-2.80845	1.72E-05	0.000647	hypothetical protein && PF01184:GPR1/FUN34/yaaH family
VP0653	VP_RS03140	-2.71623	7.47E-10	1.03E-07	molecular chaperone DnaK && PF00012:Hsp70 protein
VP0654	VP_RS03145	-1.82499	8.12E-05	0.002149	molecular chaperone DnaJ && PF00226:DnaJ domain|PF00684:DnaJ central domain|PF01556:DnaJ C terminal domain
VP0666	VP_RS03190	-1.60592	0.000172	0.003701	phosphoribosylformylglycinamidine synthase && PF13507:CobB/CobQ-like glutamine amidotransferase domain|PF02769:AIR synthase related protein, C-terminal domain
VP0671	VP_RS03215	-1.67688	0.000105	0.002656	aminoacyl-histidine dipeptidase && PF01546:Peptidase family M20/M25/M40|PF07687:Peptidase dimerisation domain
VP0695	VP_RS03335	-1.46355	0.001114	0.015562	23S rRNA (cytidine(2498)-2'-O)-methyltransferase RlmM && PF01728:FtsJ-like methyltransferase
VP0716	VP_RS03435	-1.32477	0.003401	0.034069	lipoyl synthase && PF16881:N-terminal domain of lipoyl synthase of Radical_SAM family|PF04055:Radical SAM superfamily
VP0727	VP_RS03490	-1.50089	0.0004	0.007207	leucine--tRNA ligase && PF00133:tRNA synthetases class I (I, L, M and V)|PF13603:Leucyl-tRNA synthetase, Domain 2|PF08264:Anticodon-binding domain of tRNA
VP0729	VP_RS03500	-1.3337	0.003451	0.034416	apolipoprotein N-acyltransferase && PF00795:Carbon-nitrogen hydrolase
VP0758	VP_RS03745	-1.36088	0.002806	0.029806	copper-translocating P-type ATPase && PF00122:E1-E2 ATPase|PF00403:Heavy-metal-associated domain|PF00702:haloacid dehalogenase-like hydrolase
VP0762	VP_RS03760	-1.80208	4.02E-05	0.001272	glutamate--tRNA ligase && PF00749:tRNA synthetases class I (E and Q), catalytic domain

VP0800	VP_RS03920	-1.47478	0.001537	0.019787	NAD-dependent DNA ligase LigA && PF00533:BRCA1 C Terminus (BRCT) domain|PF03120:NAD-dependent DNA ligase OB-fold domain|PF12826:Helix-hairpin-helix motif|PF01653:NAD-dependent DNA ligase adenylation domain|PF03119:NAD-dependent DNA ligase C4 zinc finger domain
VP0818	VP_RS03990	-1.79055	0.00502	0.045178	sulfite exporter TauE/SafE family protein && PF01925:Sulfite exporter TauE/SafE
VP0821	VP_RS04005	-2.23444	2.08E-06	0.000107	molecular chaperone HtpG && PF02518:Histidine kinase-, DNA gyrase B-, and HSP90-like ATPase|PF00183:Hsp90 protein
VP0850	VP_RS04145	-1.20469	0.003973	0.038243	succinate--CoA ligase subunit alpha && PF02629:CoA binding domain|PF00549:CoA-ligase
VP0903	VP_RS04400	-2.06478	6.04E-05	0.001726	DEAD/DEAH box helicase && PF00271:Helicase conserved C-terminal domain|PF00270:DEAD/DEAH box helicase
VP0905	VP_RS04410	-1.49235	0.002614	0.028374	Ktr system potassium transporter B && PF02386:Cation transport protein
VP0919	VP_RS04480	-1.94993	6.77E-06	0.000297	endopeptidase La && PF05362:Lon protease (S16) C-terminal proteolytic domain|PF00004:ATPase family associated with various cellular activities (AAA)|PF02190:ATP-dependent protease La (LON) substrate-binding domain
VP0937	VP_RS04565	-1.81141	0.00053	0.00893	MFS transporter && PF01553:Acyltransferase|PF07690:Major Facilitator Superfamily
VP0942	VP_RS04620	-2.51037	4.83E-05	0.001466	type II secretion system protein && -
VP0948	VP_RS04645	-1.89766	7.80E-05	0.002106	hypothetical protein && -
VP0956	VP_RS04690	-1.29023	0.004799	0.043814	NAD(P)/FAD-dependent oxidoreductase && -
VP0959	VP_RS04700	-1.63087	0.000133	0.003129	zinc/cadmium/mercury/lead-transporting ATPase && PF00702:haloacid dehalogenase-like hydrolase|PF00403:Heavy-metal-associated domain|PF00122:E1-E2 ATPase
VP0960	VP_RS04705	-1.29168	0.002748	0.02943	uridine phosphorylase && PF01048:Phosphorylase superfamily
VP0962	VP_RS04710	-1.4871	0.005279	0.046204	DUF1887 family protein && PF09002:Domain of unknown function (DUF1887)
VP1047	VP_RS05095	-1.22684	0.003952	0.038205	aspartate--tRNA ligase && PF02938:GAD domain|PF00152:tRNA synthetases class II (D, K and N)|PF01336:OB-fold nucleic acid binding domain
VP1070	VP_RS05210	-1.46503	0.003967	0.038243	L-alanine exporter AlaE && PF06610:L-alanine exporter

VP1099	VP_RS05350	-1.61351	0.005296	0.046204	tRNA isopentenyl-2-thiomethyl-A-37 hydroxylase MiaE && PF06175:tRNA-(MS[2]IO[6]A)-hydroxylase (MiaE)
VP1112	VP_RS05415	-2.19064	0.000974	0.014238	adenosylmethionine--8-amino-7-oxononanoate transaminase && PF00202:Aminotransferase class-III
VP1114	VP_RS05425	-1.63818	0.001646	0.020765	8-amino-7-oxononanoate synthase && PF00155:Aminotransferase class I and II
VP1115	VP_RS05430	-2.63375	7.33E-05	0.00204	malonyl-ACP O-methyltransferase BioC && PF08241:Methyltransferase domain
VP1116	VP_RS05435	-1.3892	0.004284	0.040703	ATP-dependent dethiobiotin synthetase BioD && PF13500:AAA domain
VP1185	VP_RS05760	-2.59467	1.21E-06	7.36E-05	methyl-accepting chemotaxis protein && PF00015:Methyl-accepting chemotaxis protein (MCP) signalling domain|PF00672:HAMP domain
VP1268	VP_RS06155	-1.58465	0.001019	0.014562	DUF2786 domain-containing protein && PF10979:Protein of unknown function (DUF2786)
VP1273	VP_RS06180	-1.18968	0.004941	0.04474	histidine ammonia-lyase && PF00221:Aromatic amino acid lyase
VP1274	VP_RS06185	-1.28422	0.002347	0.025979	urocanate hydratase && PF01175:Urocanase
VP1276	VP_RS06195	-1.49276	0.000728	0.011552	imidazolonepropionase && PF01979:Amidohydrolase family
VP1277	VP_RS06200	-1.65928	0.000393	0.007154	histidine utilization repressor && PF00392:Bacterial regulatory proteins, gntR family|PF07702:UTRA domain
VP1285	VP_RS06245	-1.48857	0.000646	0.010555	TetR/AcrR family transcriptional regulator && PF00440:Bacterial regulatory proteins, tetR family
VP1286	VP_RS06250	-1.99483	0.001727	0.021601	MMPL family transporter && PF03176:MMPL family
VP1302	VP_RS06325	-2.96001	3.35E-10	5.78E-08	L-cystine transporter && PF00375:Sodium:dicarboxylate symporter family
VP1332	VP_RS06470	-2.01429	0.002892	0.030438	extracellular solute-binding protein && PF13416:Bacterial extracellular solute-binding protein
VP1333	VP_RS06475	-3.95605	0.00074	0.011653	ornithine cyclodeaminase family protein && PF02423:Ornithine cyclodeaminase/mu-crystallin family
VP1422	VP_RS06910	-4.61579	1.06E-05	0.000433	SM-20 && PF13640:2OG-Fe(II) oxygenase superfamily
VP1435	VP_RS06960	-7.24251	4.78E-05	0.001466	sodium:glutamate symporter && PF03616:Sodium/glutamate symporter

VP1449	VP_RS07030	-1.94802	0.000784	0.012196	dimethyl sulfoxide reductase anchor subunit && PF04976:DMSO reductase anchor subunit (DmsC)
VP1452	VP_RS07045	-2.03505	0.000978	0.014238	FMN-dependent NADH-azoreductase && PF02525:Flavodoxin-like fold
VP1504	VP_RS07290	-1.5812	0.001146	0.015865	ABC-F family ATPase && PF00005:ABC transporter|PF12848:ABC transporter
VP1510	VP_RS07310	-1.6915	0.000215	0.004445	4Fe-4S binding protein && PF12838:4Fe-4S dicluster domain|PF13237:4Fe-4S dicluster domain
VP1511	VP_RS07315	-1.5831	0.001183	0.016044	TorD family cytoplasmic chaperone && PF02613:Nitrate reductase delta subunit
VP1513	VP_RS07325	-1.54797	0.000267	0.005322	formate dehydrogenase subunit alpha && PF01568:Molydopterin dinucleotide binding domain|PF04879:Molybdopterin oxidoreductase Fe4S4 domain|PF00384:Molybdopterin oxidoreductase
VP1514	VP_RS07330	-1.24889	0.004752	0.043596	4Fe-4S dicluster domain-containing protein && PF13247:4Fe-4S dicluster domain
VP1676	VP_RS08075	-1.69432	0.001942	0.023051	LysR family transcriptional regulator && PF00126:Bacterial regulatory helix-turn-helix protein, lysR family|PF03466:LysR substrate binding domain
VP1688	VP_RS08125	-6.252	0.004174	0.039945	HrpE/YscL family type III secretion apparatus protein && PF02108:Flagellar assembly protein FliH
VP1764	VP_RS08485	-3.84754	4.82E-10	7.11E-08	EamA family transporter && PF00892:EamA-like transporter family
VP1770	VP_RS08510	-3.42539	1.98E-06	0.000106	manganese-dependent inorganic pyrophosphatase && PF02833:DHHA2 domain|PF01368:DHH family
VP1771	VP_RS08515	-1.72707	0.000856	0.013071	4-aminobutyrate--2-oxoglutarate transaminase && PF00202:Aminotransferase class-III
VP1772	VP_RS08520	-3.34041	4.82E-07	3.33E-05	NAD-dependent succinate-semialdehyde dehydrogenase && PF00171:Aldehyde dehydrogenase family
VP1773	VP_RS08525	-2.53405	9.35E-06	0.000398	agmatine deiminase && PF04371:Porphyromonas-type peptidyl-arginine deiminase
VP1787	VP_RS08580	-2.08576	3.14E-05	0.001035	- && PF00665:Integrase core domain|PF13276:HTH-like domain
VP1873	VP_RS09125	-1.66953	8.73E-05	0.002248	fumarate hydratase && PF05681:Fumarate hydratase (Fumerase)|PF05683:Fumarase C-terminus

VP1880	VP_RS09155	-1.98168	1.85E-05	0.000684	L-serine ammonia-lyase && PF03315:Serine dehydratase beta chain|PF03313:Serine dehydratase alpha chain
VP1951	VP_RS09495	-1.6231	0.000198	0.004147	23S rRNA pseudouridine(2605) synthase RluB && PF00849:RNA pseudouridylate synthase|PF01479:S4 domain
VP1980	VP_RS09625	-1.72948	0.000129	0.003058	YgiQ family radical SAM protein && PF11842:Domain of unknown function (DUF3362)|PF08497:Radical SAM N-terminal|PF04055:Radical SAM superfamily
VP1984	VP_RS09640	-2.52299	0.004369	0.041245	HAMP domain-containing histidine kinase && PF02518:Histidine kinase-, DNA gyrase B-, and HSP90-like ATPase|PF00512:His Kinase A (phospho-acceptor) domain
VP1988	VP_RS09660	-2.51099	4.87E-05	0.001466	LysR family transcriptional regulator && PF03466:LysR substrate binding domain|PF00126:Bacterial regulatory helix-turn-helix protein, lysR family
VP2063	VP_RS10030	-1.90881	7.42E-05	0.002053	SulP family inorganic anion transporter && PF01740:STAS domain|PF00916:Sulfate permease family
VP2074	VP_RS10085	-1.89492	0.000461	0.008035	UPF0149 family protein && PF02810:SEC-C motif|PF03695:Uncharacterised protein family (UPF0149)
VP2082	VP_RS10125	-1.68399	0.000134	0.003129	acetate kinase && PF00871:Acetokinase family
VP2083	VP_RS10130	-1.35302	0.001666	0.02096	phosphate acetyltransferase && PF01515:Phosphate acetyl/butaryl transferase|PF13500:AAA domain|PF07085:DRTGG domain
VP2121	VP_RS10310	-1.35366	0.001257	0.016863	bifunctional acetaldehyde-CoA/alcohol dehydrogenase && PF00465:Iron-containing alcohol dehydrogenase|PF00171:Aldehyde dehydrogenase family
VP2157	VP_RS10480	-1.17483	0.005395	0.046936	type I glyceraldehyde-3-phosphate dehydrogenase && PF02800:Glyceraldehyde 3-phosphate dehydrogenase, C-terminal domain|PF00044:Glyceraldehyde 3-phosphate dehydrogenase, NAD binding domain
VP2160	VP_RS10495	-2.01893	0.000785	0.012196	23S rRNA (guanine(745)-N(1))-methyltransferase && PF13847:Methyltransferase domain
VP2169	VP_RS10535	-1.79038	0.001005	0.014456	YcgN family cysteine cluster protein && PF03692:Putative zinc- or iron-chelating domain
VP2249	VP_RS10920	-1.76034	6.51E-05	0.001835	flagellar basal body M-ring protein FliF && PF08345:Flagellar M-ring protein C-terminal|PF01514:Secretory protein of YscJ/FliF family

VP2251	VP_RS10930	-1.33142	0.002583	0.028174	sigma-54-dependent Fis family transcriptional regulator && PF00072:Response regulator receiver domain|PF00158:Sigma-54 interaction domain|PF02954:Bacterial regulatory protein, Fis family
VP2252	VP_RS10935	-1.61116	0.000767	0.012043	PAS domain-containing protein && PF02518:Histidine kinase-, DNA gyrase B-, and HSP90-like ATPase|PF13188:PAS domain|PF00512:His Kinase A (phospho-acceptor) domain
VP2333	VP_RS11325	-1.28319	0.002505	0.027384	proline--tRNA ligase && PF04073:Aminoacyl-tRNA editing domain|PF00587:tRNA synthetase class II core domain (G, H, P, S and T)|PF03129:Anticodon binding domain
VP2376	VP_RS11540	-3.06204	1.36E-10	2.74E-08	DUF3413 domain-containing protein && PF11893:Domain of unknown function (DUF3413)|PF00884:Sulfatase
VP2388	VP_RS11590	-2.30222	3.13E-06	0.000147	glycerol-3-phosphate dehydrogenase && PF01266:FAD dependent oxidoreductase|PF16901:C-terminal domain of alpha-glycerophosphate oxidase
VP2395	VP_RS11625	-4.95167	8.61E-07	5.53E-05	cellulase family glycosylhydrolase && PF00150:Cellulase (glycosyl hydrolase family 5)
VP2403	VP_RS11660	-1.85553	0.001161	0.016018	beta-galactosidase subunit alpha && PF02836:Glycosyl hydrolases family 2, TIM barrel domain|PF02929:Beta galactosidase small chain|PF00703:Glycosyl hydrolases family 2|PF16353:Domain of unknown function(DUF4981)|PF02837:Glycosyl hydrolases family 2, sugar binding domain
VP2428	VP_RS11775	-1.21702	0.003492	0.034748	elongation factor G && PF03144:Elongation factor Tu domain 2|PF14492:Elongation Factor G, domain II|PF00679:Elongation factor G C-terminus|PF03764:Elongation factor G, domain IV|PF00009:Elongation factor Tu GTP binding domain
VP2449	VP_RS11880	-2.37766	2.41E-07	1.84E-05	sodium-coupled multidrug efflux MATE transporter VmrA && PF01554:MatE
VP2452	VP_RS11895	-1.59246	0.000159	0.003528	polyribonucleotide nucleotidyltransferase && PF00575:S1 RNA binding domain|PF00013:KH domain|PF03725:3' exoribonuclease family, domain 2|PF03726:Polyribonucleotide nucleotidyltransferase, RNA binding domain|PF01138:3' exoribonuclease family, domain 1

VP2457	VP_RS11920	-1.19051	0.004771	0.043649	transcription termination/antitermination protein NusA && PF08529:NusA N-terminal domain|PF14520:Helix-hairpin-helix domain|PF13184:NusA-like KH domain
VP2480	VP_RS12040	-2.47437	0.002142	0.024262	ABC transporter permease && PF00528:Binding-protein-dependent transport system inner membrane component
VP2482	VP_RS12050	-1.83721	0.001104	0.015532	ABC transporter ATP-binding protein && PF08352:Oligopeptide/dipeptide transporter, C-terminal region|PF00005:ABC transporter
VP2488	VP_RS12080	-1.59834	0.00205	0.023705	phosphoglucomutase/phosphomannomutase family protein && PF02879:Phosphoglucomutase/phosphomannomutase, alpha/beta/alpha domain II|PF02878:Phosphoglucomutase/phosphomannomutase, alpha/beta/alpha domain I|PF00408:Phosphoglucomutase/phosphomannomutase, C-terminal domain|PF02880:Phosphoglucomutase/phosphomannomutase, alpha/beta/alpha domain III
VP2517	VP_RS12205	-1.16348	0.005415	0.047012	dihydrolipoyl dehydrogenase && PF02852:Pyridine nucleotide-disulphide oxidoreductase, dimerisation domain|PF07992:Pyridine nucleotide-disulphide oxidoreductase
VP2518	VP_RS12210	-1.46273	0.000549	0.009137	pyruvate dehydrogenase complex dihydrolipoyllysine-residue acetyltransferase && PF02817:e3 binding domain|PF00364:Biotin-requiring enzyme|PF00198:2-oxoacid dehydrogenases acyltransferase (catalytic domain)
VP2519	VP_RS12215	-2.06093	1.69E-06	9.61E-05	pyruvate dehydrogenase (acetyl-transferring)%2C homodimeric type && PF00456:Transketolase, thiamine diphosphate binding domain
VP2520	VP_RS12220	-2.18448	2.44E-06	0.00012	pyruvate dehydrogenase complex transcriptional repressor PdhR && PF07729:FCD domain|PF00392:Bacterial regulatory proteins, gntR family
VP2534	VP_RS12290	-1.18606	0.00501	0.045178	signal recognition particle protein && PF02978:Signal peptide binding domain|PF02881:SRP54-type protein, helical bundle domain|PF00448:SRP54-type protein, GTPase domain
VP2550	VP_RS12415	-1.34243	0.001613	0.02052	recombinase RecA && PF00154:recA bacterial DNA recombination protein
VP2570	VP_RS12510	-1.66214	0.002336	0.025923	DNA repair protein RecO && PF11967:Recombination protein O N terminal|PF02565:Recombination protein O C terminal

VP2574	VP_RS12530	-1.62374	0.000151	0.003455	elongation factor 4 && PF00679:Elongation factor G C-terminus|PF06421:GTP-binding protein LepA C-terminus|PF03144:Elongation factor Tu domain 2|PF00009:Elongation factor Tu GTP binding domain
VP2575	VP_RS12535	-1.8362	0.000109	0.002747	SoxR reducing system RseC family protein && PF04246:Positive regulator of sigma(E), RseC/MucC
VP2596	VP_RS12630	-2.73163	8.25E-05	0.002154	amino acid transporter && PF01810:LysE type translocator
VP2605	VP_RS12670	-1.68799	0.000181	0.003832	transketolase && PF02780:Transketolase, C-terminal domain|PF00456:Transketolase, thiamine diphosphate binding domain|PF02779:Transketolase, pyrimidine binding domain
VP2611	VP_RS12700	-1.81463	0.000115	0.002825	glutathione synthase && PF02951:Prokaryotic glutathione synthetase, N-terminal domain|PF02955:Prokaryotic glutathione synthetase, ATP-grasp domain
VP2646	VP_RS12980	-1.39397	0.000976	0.014238	valine--tRNA ligase && PF08264:Anticodon-binding domain of tRNA|PF10458:Valyl tRNA synthetase tRNA binding arm|PF00133:tRNA synthetases class I (I, L, M and V)
VP2718	VP_RS13330	-1.67969	0.000248	0.005028	sodium:proton antiporter && PF00999:Sodium/hydrogen exchanger family
VP2722	VP_RS13375	-1.54533	0.000301	0.005802	assimilatory sulfite reductase (NADPH) flavoprotein subunit && PF00175:Oxidoreductase NAD-binding domain|PF00667:FAD binding domain|PF00258:Flavodoxin
VP2762	VP_RS13560	-1.7979	6.16E-05	0.001749	PadR family transcriptional regulator && PF10400:Virulence activator alpha C-term|PF03551:Transcriptional regulator PadR-like family
VP2804	VP_RS13745	-1.257	0.004274	0.040699	tryptophan--tRNA ligase && PF00579:tRNA synthetases class I (W and Y)
VP2812	VP_RS13780	-1.31857	0.00177	0.021888	adenylosuccinate synthase && PF00709:Adenylosuccinate synthetase
VP2829	VP_RS13930	-1.86437	2.01E-05	0.000735	2%2C3-bisphosphoglycerate-independent phosphoglycerate mutase && PF01676:Metalloenzyme superfamily|PF06415:BPG-independent PGAM N-terminus (iPGM_N)
VP2844	VP_RS14000	-2.12815	3.66E-06	0.000167	hypothetical protein && PF01145:SPFH domain / Band 7 family
VP2851	VP_RS14030	-2.30956	1.70E-07	1.45E-05	chaperonin GroEL && PF00118:TCP-1/cpn60 chaperonin family
VP2852	VP_RS14035	-1.53041	0.003832	0.037296	co-chaperone GroES && -
VP2901	VP_RS14315	-2.43224	4.39E-07	3.09E-05	hypothetical protein && -

VP2943	VP_RS14575	-2.01239	0.00117	0.016044	MATE family efflux transporter DinF && PF01554:MatE
VP2945	VP_RS14585	-1.2091	0.005124	0.045655	repressor LexA && PF00717:Peptidase S24-like|PF01726:LexA DNA binding domain
VP2987	VP_RS14785	-1.56315	0.000269	0.005342	class I adenylate cyclase && PF01295:Adenylate cyclase, class-I|PF12633:Adenylate cyclase NT domain
VP3004	VP_RS14880	-1.64917	0.001835	0.022418	7-cyano-7-deazaguanine/7-aminomethyl-7-deazaguanine transporter && PF02592:Putative vitamin uptake transporter
VP3013	VP_RS14915	-1.25334	0.004959	0.044815	DNA helicase II && PF13361:UvrD-like helicase C-terminal domain|PF00580:UvrD/REP helicase N-terminal domain
VP3020	VP_RS14950	-1.89981	0.002384	0.026197	LysR family transcriptional regulator && PF03466:LysR substrate binding domain|PF00126:Bacterial regulatory helix-turn-helix protein, lysR family
VP3025	VP_RS14975	-2.90076	0.004849	0.04409	thiazole biosynthesis adenylyltransferase ThiF && PF00899:ThiF family
VP3026	VP_RS14980	-2.22223	0.002817	0.029846	thiamine phosphate synthase && PF02581:Thiamine monophosphate synthase/TENI
VP3043	VP_RS15095	-1.72423	0.000539	0.009035	methionyl-tRNA formyltransferase && PF02911:Formyl transferase, C-terminal domain|PF00551:Formyl transferase
VP3058	VP_RS15165	-1.20284	0.004751	0.043596	acetolactate synthase 2 catalytic subunit && PF02775:Thiamine pyrophosphate enzyme, C-terminal TPP binding domain|PF00205:Thiamine pyrophosphate enzyme, central domain|PF02776:Thiamine pyrophosphate enzyme, N-terminal TPP binding domain
VPA0031	VP_RS15400	-1.47645	0.000776	0.012143	sodium/glutamate symporter && PF03616:Sodium/glutamate symporter
VPA0097	VP_RS15715	-1.53332	0.001043	0.014806	HlyD family secretion protein && PF13533:Biotin-lipoyl like|PF13437:HlyD family secretion protein
VPA0102	VP_RS15740	-1.59682	0.000212	0.004399	alpha-glucosidase && -
VPA0168	VP_RS16095	-3.89333	2.42E-13	6.70E-11	multidrug efflux MFS transporter EmrD && PF07690:Major Facilitator Superfamily
VPA0256	VP_RS16505	-2.49056	1.92E-07	1.55E-05	hypothetical protein && -
VPA0287	VP_RS16645	-1.75316	0.000103	0.002625	chaperonin GroEL && PF00118:TCP-1/cpn60 chaperonin family
VPA0307	VP_RS16750	-2.6262	1.80E-06	9.86E-05	nicotinamidase && PF00857:Isochorismatase family
VPA0308	VP_RS16755	-1.52348	0.002384	0.026197	NUDIX hydrolase && PF00293:NUDIX domain

VPA0309	VP_RS16760	-2.88988	5.79E-08	5.46E-06	nicotinate phosphoribosyltransferase && PF04095:Nicotinate phosphoribosyltransferase (NAPRTase) family
VPA0387	VP_RS17095	-2.11416	0.004621	0.042897	LysR family transcriptional regulator && PF03466:LysR substrate binding domain|PF00126:Bacterial regulatory helix-turn-helix protein, lysR family
VPA0390	VP_RS17110	-1.9286	0.000471	0.008052	DEAD/DEAH box helicase && PF00270:DEAD/DEAH box helicase|PF00271:Helicase conserved C-terminal domain
VPA0406	VP_RS17185	-1.55184	0.002744	0.02943	DUF808 domain-containing protein && PF05661:Protein of unknown function (DUF808)
VPA0452	VP_RS17400	-1.62653	0.002894	0.030438	cytochrome b && PF00033:Cytochrome b/b6/petB
VPA0471	VP_RS17490	-2.28848	0.001999	0.023515	efflux RND transporter permease subunit VmeQ && PF00873:AcrB/AcrD/AcrF family
VPA0482	VP_RS17545	-7.10596	0.00012	0.0029	TolC family protein && PF02321:Outer membrane efflux protein
VPA0486	VP_RS17560	-2.14037	0.002025	0.023658	endonuclease/exonuclease/phosphatase family protein && PF03372:Endonuclease/Exonuclease/phosphatase family
VPA0491	VP_RS17585	-2.96448	0.003385	0.034069	methyl-accepting chemotaxis protein && PF00015:Methyl-accepting chemotaxis protein (MCP) signalling domain|PF00672:HAMP domain|PF12729:Four helix bundle sensory module for signal transduction
VPA0500	VP_RS17625	-2.56145	0.001185	0.016044	PTS transporter subunit EIIA && PF00359:Phosphoenolpyruvate-dependent sugar phosphotransferase system, EIIA 2
VPA0501	VP_RS17630	-3.53048	3.90E-10	5.95E-08	PTS mannitol transporter subunit IICB && PF02302:PTS system, Lactose/Cellobiose specific IIB subunit|PF02378:Phosphotransferase system, EIIC
VPA0502	VP_RS17635	-2.46157	2.15E-06	0.000109	zinc-binding dehydrogenase && PF00107:Zinc-binding dehydrogenase|PF08240:Alcohol dehydrogenase GroES-like domain
VPA0526	VP_RS17740	-3.05786	1.50E-09	2.01E-07	porin && PF13609:Gram-negative porin
VPA0572	VP_RS17955	-1.25631	0.003002	0.031204	cation diffusion facilitator family transporter && PF01545:Cation efflux family
VPA0590	VP_RS18040	-1.41615	0.003705	0.036297	DEAD/DEAH box helicase && PF00270:DEAD/DEAH box helicase|PF00271:Helicase conserved C-terminal domain

VPA0608	VP_RS18125	-1.31557	0.001894	0.022604	DEAD/DEAH box helicase && PF00270:DEAD/DEAH box helicase|PF00271:Helicase conserved C-terminal domain|PF03880:DbpA RNA binding domain
VPA0612	VP_RS18140	-2.08494	5.45E-05	0.001597	PAS domain S-box protein && PF00015:Methyl-accepting chemotaxis protein (MCP) signalling domain|PF08447:PAS fold|PF13426:PAS domain
VPA0628	VP_RS18220	-1.67519	0.000167	0.003631	cytochrome o ubiquinol oxidase subunit I && PF00115:Cytochrome C and Quinol oxidase polypeptide I
VPA0655	VP_RS18335	-2.1269	0.000161	0.003556	hypothetical protein && -
VPA0683	VP_RS18465	-3.54392	0.005247	0.046204	arylsulfatase && PF00884:Sulfatase|PF14707:C-terminal region of aryl-sulfatase
VPA0694	VP_RS18520	-2.19064	0.000974	0.014238	YgiW/YdeI family stress tolerance OB fold protein && PF04076:Bacterial OB fold (BOF) protein
VPA0695	VP_RS18525	-2.90076	0.004849	0.04409	porin family protein && PF13505:Outer membrane protein beta-barrel domain
VPA0712	VP_RS18600	-1.35659	0.002992	0.031171	ribosome biogenesis GTPase YlqF && PF01926:50S ribosome-binding GTPase
VPA0747	VP_RS18765	-6.78664	0.000517	0.008776	type II secretion system protein && PF13544:Type IV pilin N-term methylation site GFxxxE
VPA0755	VP_RS18795	-2.28266	0.005286	0.046204	peptidase && PF03413:Peptidase propeptide and YPEB domain|PF04151:Bacterial pre-peptidase C-terminal domain|PF02868:Thermolysin metallopeptidase, alpha-helical domain|PF07504:Fungalysin/Thermolysin Propeptide Motif|PF01447:Thermolysin metallopeptidase, catalytic domain
VPA0778	VP_RS18905	-2.40665	0.001859	0.022456	magnesium transporter && PF03448:MgtE intracellular N domain|PF01769:Divalent cation transporter
VPA0801	VP_RS19015	-1.5079	0.000371	0.006787	aminomethyl-transferring glycine dehydrogenase && PF02347:Glycine cleavage system P-protein
VPA0803	VP_RS19025	-1.40004	0.000997	0.014422	serine hydroxymethyltransferase && PF00464:Serine hydroxymethyltransferase
VPA0805	VP_RS19035	-1.76331	4.58E-05	0.001418	glycine cleavage system aminomethyltransferase GcvT && PF08669:Glycine cleavage T-protein C-terminal barrel domain|PF01571:Aminomethyltransferase folate-binding domain

VPA0806	VP_RS19040	-1.33039	0.005064	0.045387	efflux transporter transcriptional repressor VdeR && PF00440:Bacterial regulatory proteins, tetR family
VPA0807	VP_RS19045	-4.7155	1.10E-15	6.37E-13	multidrug efflux RND transporter periplasmic adaptor subunit VmeT && PF13533:Biotin-lipoyl like|PF13437:HlyD family secretion protein
VPA0808	VP_RS19050	-4.48893	2.72E-14	9.27E-12	multidrug efflux RND transporter periplasmic adaptor subunit VmeU && PF13533:Biotin-lipoyl like
VPA0809	VP_RS19055	-4.03772	1.24E-16	1.10E-13	multidrug efflux RND transporter permease subunit VmeV && PF00873:AcrB/AcrD/AcrF family
VPA0813	VP_RS19075	-1.72702	0.004015	0.038566	fused PTS fructose transporter subunit IIA/HPr protein && PF00359:Phosphoenolpyruvate-dependent sugar phosphotransferase system, EIIA 2|PF00381:PTS HPr component phosphorylation site
VPA0830	VP_RS19155	-1.86495	0.002215	0.024898	AraC family transcriptional regulator && PF12833:Helix-turn-helix domain|PF06719:AraC-type transcriptional regulator N-terminus
VPA0832	VP_RS19160	-1.35748	0.0034	0.034069	chitinase && PF00704:Glycosyl hydrolases family 18|PF02839:Carbohydrate binding domain|PF06483:Chitinase C|PF14600:Cellulose-binding domain
VPA0835	VP_RS19175	-2.17352	1.26E-05	0.000509	inosine/guanosine kinase && PF00294:pfkB family carbohydrate kinase
VPA0845	VP_RS19210	-1.56411	0.001748	0.021748	DEAD/DEAH box helicase && PF00270:DEAD/DEAH box helicase|PF00271:Helicase conserved C-terminal domain
VPA0865	VP_RS19325	-1.7922	0.001181	0.016044	DUF2238 domain-containing protein && PF09997:Predicted membrane protein (DUF2238)
VPA0933	VP_RS19645	-2.89035	3.35E-06	0.000155	NirD/YgiW/YdeI family stress tolerance protein && PF04076:Bacterial OB fold (BOF) protein
VPA0946	VP_RS19705	-3.57937	1.46E-06	8.64E-05	YeeE/YedE family protein && PF04143:Sulphur transport
VPA0947	VP_RS19710	-5.36561	1.77E-08	1.82E-06	helix-turn-helix transcriptional regulator && PF12840:Helix-turn-helix domain
VPA0949	VP_RS19720	-1.22206	0.004677	0.043322	pyridine nucleotide-disulfide oxidoreductase family protein && PF00581:Rhodanese-like domain|PF07992:Pyridine nucleotide-disulphide oxidoreductase|PF02852:Pyridine nucleotide-disulphide oxidoreductase, dimerisation domain

VPA0959	VP_RS19760	-1.38223	0.003263	0.03329	FdhF/YdeP family oxidoreductase && PF01568:Molydopterin dinucleotide binding domain|PF00384:Molybdopterin oxidoreductase
VPA0973	VP_RS19825	-2.55903	4.24E-07	3.03E-05	MFS transporter && PF07690:Major Facilitator Superfamily
VPA1009	VP_RS20015	-1.6474	0.000353	0.006532	DUF3763 domain-containing protein && PF12592:Protein of unknown function (DUF3763)|PF07728:AAA domain (dynein-related subfamily)
VPA1051	VP_RS20220	-2.87908	9.24E-07	5.76E-05	glutathione S-transferase && PF13417:Glutathione S-transferase, N-terminal domain|PF00043:Glutathione S-transferase, C-terminal domain
VPA1053	VP_RS20230	-1.84041	0.002045	0.023705	DUF296 domain-containing protein && PF03479:Domain of unknown function (DUF296)
VPA1077	VP_RS20335	-1.60411	0.002142	0.024262	hypothetical protein && -
VPA1166	VP_RS20755	-1.87299	0.001005	0.014456	H(+)/Cl(-) exchange transporter ClcA && PF00654:Voltage gated chloride channel
VPA1168	VP_RS20765	-4.06087	2.69E-15	1.19E-12	divalent metal cation transporter && PF01566:Natural resistance-associated macrophage protein
VPA1209	VP_RS20965	-2.29658	1.47E-05	0.000571	protein translocase subunit SecD && PF02355:Protein export membrane protein|PF13721:SecD export protein N-terminal TM region
VPA1218	VP_RS21010	-2.0755	0.001866	0.022456	HlyD family secretion protein && PF13533:Biotin-lipoyl like
VPA1227	VP_RS21050	-1.60779	0.002266	0.025339	MATE family efflux transporter && PF01554:MatE
VPA1280	VP_RS21300	-2.22123	1.27E-05	0.000509	phosphoethanolamine--lipid A transferase && PF08019:Domain of unknown function (DUF1705)|PF00884:Sulfatase
VPA1286	VP_RS21325	-1.51779	0.005142	0.045719	MarR family transcriptional regulator && PF01047:MarR family
VPA1298	VP_RS21385	-1.68267	0.000465	0.008052	glutathione-dependent disulfide-bond oxidoreductase && PF02798:Glutathione S-transferase, N-terminal domain|PF13410:Glutathione S-transferase, C-terminal domain
VPA1306	VP_RS21420	-2.57925	0.004369	0.041245	metal ABC transporter permease && PF00950:ABC 3 transport family
VPA1308	VP_RS21430	-3.0868	3.15E-05	0.001035	hypothetical protein && -
VPA1415	VP_RS21930	-1.47012	0.001463	0.018941	TetR/AcrR family transcriptional regulator && PF16925:Bacterial transcriptional repressor C-terminal|PF00440:Bacterial regulatory proteins, tetR family

VPA1416	VP_RS21935	-4.03987	1.66E-08	1.75E-06	NADP-dependent oxidoreductase && PF16884:N-terminal domain of oxidoreductase|PF00107:Zinc-binding dehydrogenase
VPA1417	VP_RS21940	-1.57722	0.00474	0.043596	glutathione S-transferase && PF00043:Glutathione S-transferase, C-terminal domain|PF02798:Glutathione S-transferase, N-terminal domain
VPA1468	VP_RS22190	-1.60327	0.000737	0.011648	DEAD/DEAH box helicase && PF00270:DEAD/DEAH box helicase|PF00271:Helicase conserved C-terminal domain
VPA1492	VP_RS22300	-1.822	0.000523	0.008843	methyl-accepting chemotaxis protein && PF00015:Methyl-accepting chemotaxis protein (MCP) signalling domain|PF00672:HAMP domain
VPA1498	VP_RS22330	-1.30722	0.002594	0.028223	L-lactate permease && PF02652:L-lactate permease
VPA1509	VP_RS22380	-1.63361	0.000153	0.003477	L-threonine 3-dehydrogenase && PF08240:Alcohol dehydrogenase GroES-like domain|PF00107:Zinc-binding dehydrogenase
VPA1510	VP_RS22385	-1.65584	0.000141	0.003259	glycine C-acetyltransferase && PF00155:Aminotransferase class I and II
VPA1586	VP_RS22745	-1.40255	0.002324	0.025853	ferredoxin--NADP reductase && PF00970:Oxidoreductase FAD-binding domain|PF00175:Oxidoreductase NAD-binding domain
VPA1682	VP_RS23205	-2.71225	0.000315	0.006047	MarR family transcriptional regulator && PF01047:MarR family
VPA1700	VP_RS23285	-3.84537	1.16E-07	1.03E-05	mannonate dehydratase && PF03786:D-mannonate dehydratase (UxuA)
VPA1702	VP_RS23295	-2.24683	0.002639	0.028571	TRAP transporter substrate-binding protein && PF03480:Bacterial extracellular solute-binding protein, family 7
VPA1751	VP_RS23520	-1.49188	0.000934	0.013929	ParB/RepB/Spo0J family partition protein && PF02195:ParB-like nuclease domain

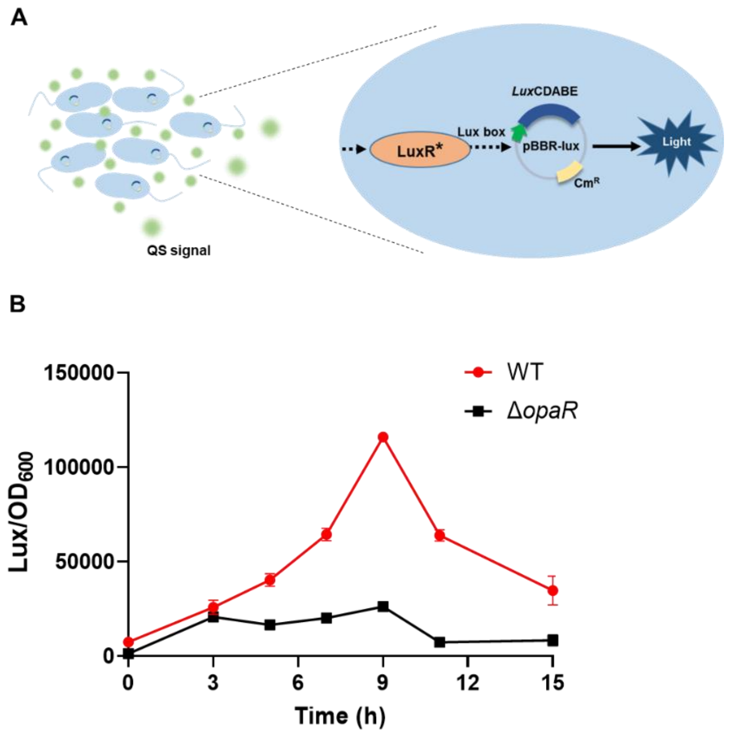

Supplemental Figure S1 The reporter plasmid pBBR-*lux* checked QS status in *V. parahaemolyticus*. (A) A working model of the pBBR-*lux* reporter. The reporter plasmid pBBR-*lux* was constructed based on bioluminescence *lux*CDABE operon of *V. harveyi*. When *V. parahaemolyticus* detect the external signals, the LuxR-type regulators will be activated and modulates the bacterial QS status. LuxR-types regulators then stimulate the transcription of *luxCDABE* to cause bioluminescence by interacting with the Lux box on pBBR-*lux*. (B) The luminescence was measured in the Δ*opaR* and WT strain with the pBBR-*lux* reporter. All data were shown as means ± SD from three replicates.

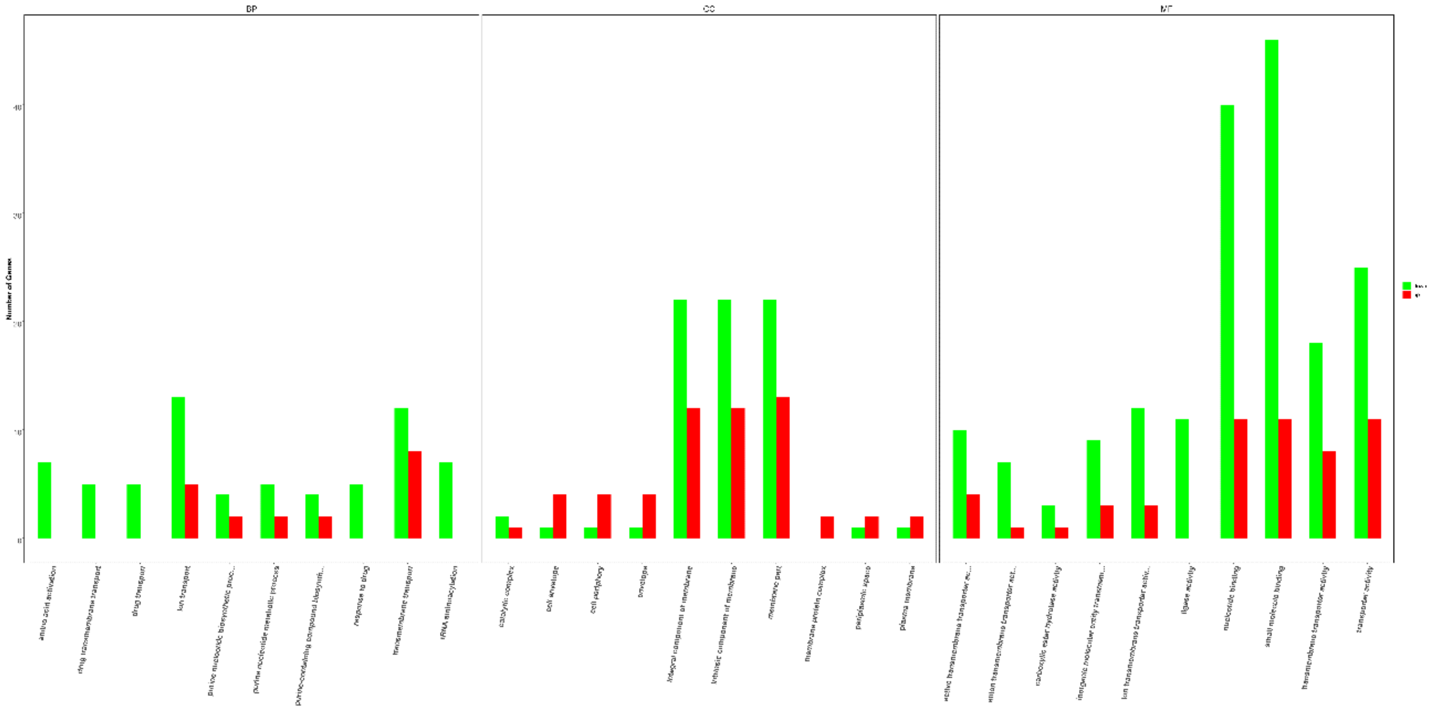

Supplemental Figure S2 GO analysis of differentially expressed proteins. These proteins are grouped into three hierarchically structured terms: biological process, cellular component, and molecular function

References

Hanahan, D. (1983). Studies on transformation of Escherichia coli with plasmids. *J Mol Biol* 166(4), 557-580. doi: 10.1016/s0022-2836(83)80284-8.

Karimova, G., Pidoux, J., Ullmann, A., and Ladant, D. (1998). A bacterial two-hybrid system based on a reconstituted signal transduction pathway. *Proc Natl Acad Sci U S A* 95(10), 5752-5756. doi: 10.1073/pnas.95.10.5752.

Philippe, N., Alcaraz, J.P., Coursange, E., Geiselmann, J., and Schneider, D. (2004). Improvement of pCVD442, a suicide plasmid for gene allele exchange in bacteria. *Plasmid* 51(3), 246-255. doi: 10.1016/j.plasmid.2004.02.003.

Yu, Y., Fang, L., Zhang, Y., Sheng, H., and Fang, W. (2015). VgrG2 of type VI secretion system 2 of Vibrio parahaemolyticus induces autophagy in macrophages. *Front Microbiol* 6, 168. doi: 10.3389/fmicb.2015.00168.
